# Effects of long-term wastewater irrigation on microplastics pollution in agricultural soil

**DOI:** 10.1007/s11356-025-36452-x

**Published:** 2025-04-29

**Authors:** Senar Aydin, Osman Mucevher, Arzu Ulvi, Fatma Beduk, Mehmet Emin Aydin, Ozen Merken, Cihan Uzun

**Affiliations:** 1 Department of Environmental Engineering, Necmettin Erbakan University, Konya, Türkiye; 2 Ministry of Agriculture and Forestry, Konya Soil, Water and Deserting Control Research Institute, Konya, Türkiye; 3 Department of Civil Engineering, Necmettin Erbakan University, Konya, Türkiye; 4 Ministry of Agriculture and Forestry, Olive Research Institute, İzmir, Türkiye

**Keywords:** Microplastics, Soil, Wastewater irrigation

## Abstract

Microplastic (MP) pollution in agroecosystems is a growing concern with unknown consequences for sustainable agricultural activities. Few studies have revealed MPs in soil as a result of wastewater irrigation, despite the increasing application of wastewater irrigation and inadequacy of conventional wastewater treatment plants in removing microplastics (MPs). In this study, the effect of treated wastewater (TWW) irrigation on MPs accumulation in agricultural soils of Konya City (in Türkiye) and the potential risks on agricultural ecosystem were investigated. For this purpose, 202 soil samples taken from 90 TWW irrigated lands and 11 non-agricultural control lands, at depths of 0–10 cm and 10–20 cm, were analyzed for color, shape, and polymer type. The risk level of MPs pollution was determined by the pollution factor (CF), pollution load index (PLI), and polymer risk index (H). The relationship between some physico-chemical properties of the soil and MPs pollution level was also analyzed. The findings of this study revealed a significant difference (P < 0.0001) in the MP count in the TWW irrigated soils, and control soils. While the average numbers of MPs for control soils were 169 ± 46.8 MPs/kg (100–220 MPs/kg) and 140 ± 44.7 MPs/kg (80–240 MPs/kg) for the 0–10 cm and 10–20 cm soil depths, respectively; 329 ± 139.5 MPs/kg (100–840 MPs/kg) and 295 ± 115.4 MPs/kg (80–660 MPs/kg) were identified for TWW irrigated soil samples taken from the same soil depths. Fiber, film, and fragment type MPs were found to be dominant polymer types in TWW irrigated soil, with 56%, 23%, and 16%, respectively. Transparent colored MPs were predominant. MP decreased from 0–10 cm to 10–20 cm depths. Most of the samples were significantly contaminated with MPs (3 ≤ CF < 6), categorized in hazard category class I (PLI < 10). The findings of this study indicate that TWW irrigation increases the accumulation of MPs in agricultural soils, which poses a higher risk to more fertile soils with higher organic matter, total nitrogen, and available phosphorus content. Hazard index assessments reveal that the soils of Konya, often referred to as the "granary of Türkiye," are at risk of MPs contamination. The findings showed that MPs, a neglected type of pollution for soil, will become an even more important problem with increasing wastewater irrigation.

## Introduction

The growing consumption of plastic products in recent decades, and insufficient management of plastic wastes has resulted in plastic pollution in agroecosystem (Guo et al. 2020; Zhang et al. 2022). Major sources of MPs in soil are wastewater irrigation, soil mulching, and soil amendment with sewage sludge and compost (Qi et al. 2020; Corradini et al. 2019; Blasing and Amelung 2018). Ultraviolet radiation, abrasion from collisions, hydrolysis, soil erosion, and biological effects cause plastics to break down into MPs, which are smaller than 5 mm in particle size (Chen et al. 2022a). MPs are formed in different shapes including fiber, fragment, film, foam, pellet, with different polymer components including polyethylene (PE), high-density polyethylene (HDPE), low-density polyethylene (LDPE), polyethylene terephthalate (PET), polyvinyl chloride (PVC), polypropylene (PP), polycarbonate (PC), polyamide (PA), and polystyrene (PS), and in various colors (Siddiqui et al. 2008).

While many studies have been conducted on MPs pollution in the aquatic environments, the terrestrial areas have attracted less attention in this context. It was first suggested by Rilling (2012) that MPs could harm the soil environment. Nizzetto et al. (2016) reported that the annual release of MPs into the soil is 4 to 23 times greater than that released into the oceans. MPs have a strong adsorption capacity due to their small size and large specific surface area. They adsorb and carry many toxic chemicals, such as heavy metals, pharmaceuticals, and persistent organic pollutants (Fu et al. 2021; Zhang et al. 2020).

Agricultural sustainability is under pressure due to various factors such as climate change, water scarcity, the use of fertile land for non-agricultural purposes, and soil infertility. The decreasing availability of clean water sources and the nutrients present in wastewater motivate farmers to use treated or untreated wastewater for agricultural irrigation. However, wastewater contains high levels of MPs. Although a high percentage of MPs are removed in wastewater treatment plants (WWTPs), a significant portion is released into the environment through wastewater discharges. Gatidou et al. (2019) detected MPs in the influent of WWTPs in Europe, America, and Austria at concentrations ranging from 1 to 3,163 MP/L, and in the effluent at concentrations ranging from 0.0007 to 125 MP/L. MPs originating from polymer fibers released during the washing of textile fabrics, microbeads from personal care and hygiene products (such as shampoo, toothpaste, and exfoliants), and MPs from automotive tires reach the sewage system and WWTPs. Conventional WWTPs are insufficient for the complete removal of MPs. Gies et al. (2018) reported that despite a MP removal efficiency of 97–99% at Vancouver’s largest WWTP, approximately 30 billion MPs are discharged into the environment annually. The repeated irrigation of soils with insufficiently treated wastewater and amendment with sewage sludge lead to the formation of MP pollution in the soil. MPs have been detected in domestic wastewater at concentrations of up to 627,000 MP/m^3^. The amounts of PE and PP in the wastewater have been identified in the range of 80–260 mg/m^3^ (He et al. 2018).

WWTP sludge is also used for soil amendment due to its positive effects on agricultural productivity. However, MPs accumulate in the sewage sludge during the treatment process. Li et al. (2018) detected MPs in sewage sludge samples from 28 WWTPs in 11 provinces in China, with concentrations ranging from 1.60 to 564 × 10^3^ MP/kg dry sludge. It was estimated that after five applications of sludge (200 dry tons/ha), the average concentration of MPs in the soil could reach 3.5 MP/g in a year (Corradini et al. 2019). According to the findings of another study conducted in China’s intensively farmed areas, soils receiving approximately 23 tons of sludge per hectare per year had MPs concentrations ranging from 7 to 43 MP/g (Zhang and Liu 2018).

MPs can lead to changes in the soil’s structure, nutrient availability, water retention capacity, and microbial activity by altering its physical, chemical, and biological properties (Zhou et al. 2021). One of the most significant effects of MPs in soil is their ability to alter soil structure, including bulk density, aggregate stability, and water retention capacity (de Souza Machado et al. 2019; Rillig and Lehmann 2020; Wang et al. 2022). MPs can disrupt soil aggregation by binding soil minerals and organic components (Rillig et al. 2017; Lei et al. 2018). The presence of MP films reduces the abundance and diversity of actinobacteria, one of the most important bacterial groups contributing to soil aggregation (Lehmann et al. 2017). They can affect soil enzyme activities, changing the soil’s nutrient and geochemical cycles. Since micro- and macro-organisms play an important role in material circulation and energy transfer in the soil, adverse effects on soil organisms result in a decrease in soil fertility (Yi et al. 2021). MPs can penetrate the intestinal walls of nematodes, cause oxidative stress, and affect gene distribution (Zhu et al. 2018). It was found that earthworms accidentally ingesting MPs lead to false satiety, reduce normal feeding behavior, and thus hinder their normal growth and reproduction (Baeza et al. 2020). Plastics are generally less dense than soil minerals, and the presence of MPs can lead to a reduction in the soil’s bulk density (de Souza Machado et al. 2018). This reduction has the potential to increase soil aeration, which can facilitate root penetration (Khan et al. 2024). On the other hand, the hydrophobicity of MPs may result in less water storage in smaller pores, which can affect hydraulic conductivity and nutrient transport in the soil. Overall, it was reported that most MPs can trigger cracking on the soil surface and significantly alter soil moisture (Jiang et al. 2017). Besides, MPs may also alter soil pH to make it more acidic or alkaline. It has been found that HDPE reduces soil pH after exposure to light and heat (Boots et al. 2019).

In soil ecosystems, plants represent the starting point of bioaccumulation in the food chain (Yin et al. 2021). Different parts of the plants have varying degrees of MPs absorption due to differences in surface charges (Bosker et al. 2019). Studies have reported the presence of MPs in the rhizomes of fruits and vegetables, such as *Triticum aestivum, Lactuca sativa, Allium fistulosum, Vicia faba*, and *Arabidopsis thaliana* (Qi et al. 2018; Li et al. 2021). According to findings of a study about the ecotoxicity and genotoxicity of PS for broad bean (*Vicia faba*), a significant inhibitory effect on the growth of *Vicia faba* were observed, as MPs blocked cell wall pores and potentially disrupted cell connections for nutrient transport (Jiang et al. 2019). It has also been reported that biodegradable mulch film has adverse effects on cultivated crops, such as wheat (Qi et al. 2018).

MPs contamination in terrestrial environments has been less studied than aquatic bodies. There are only a few recent studies on the identification of MPs in soil as a result of TWW irrigation. Although there are many studies on the inadequacy of conventional WWTPs in removing MPs from wastewater, the issue of MPs accumulation in soil after irrigation with effluents of WWTPs and its consequences for agricultural sustainability is still an unclear topic. The agricultural lands of Konya Province have significant agricultural potential in Türkiye due to cereal cultivation. The region is at risk in terms of the sustainability of agriculture due to drought. This situation encourages farmers to irrigate with TWW. Therefore, Konya Province is a suitable area for researching MPs pollution caused by TWW irrigation. In this context, the present study has been carried out to determine the effect of MPs pollution from long-term TWW irrigation in the soil of agricultural lands of Konya Province. This study is the first to evaluate the potential risk levels of MPs, as a consequence of TWW irrigation.

## Materials and methods

### Study area and sampling

Soil samples were collected along the main drainage channel, receiving the effluents of Konya urban WWTP in Türkiye. The Konya main drainage channel was constructed in 1974. This channel is approximately 150 km long, with a maximum discharge capacity of 25 m^3^/s, and it flows into Salt Lake. WWTP was constructed in 2010. The untreated urban wastewater was used for agricultural irrigation until 2010. Since then, the TWW has continued to be used for agricultural irrigation purposes in the same manner, even though it was forbidden by the state. Sampling was performed in fall 2023. In this season, while some fields were being prepared for planting, there were also fields with crops that had been planted and were awaiting harvest. Crops, such as wheat, barley, oats, alfalfa, and sunflower, have been cultivated in this agricultural area.

**Fig. 1:**
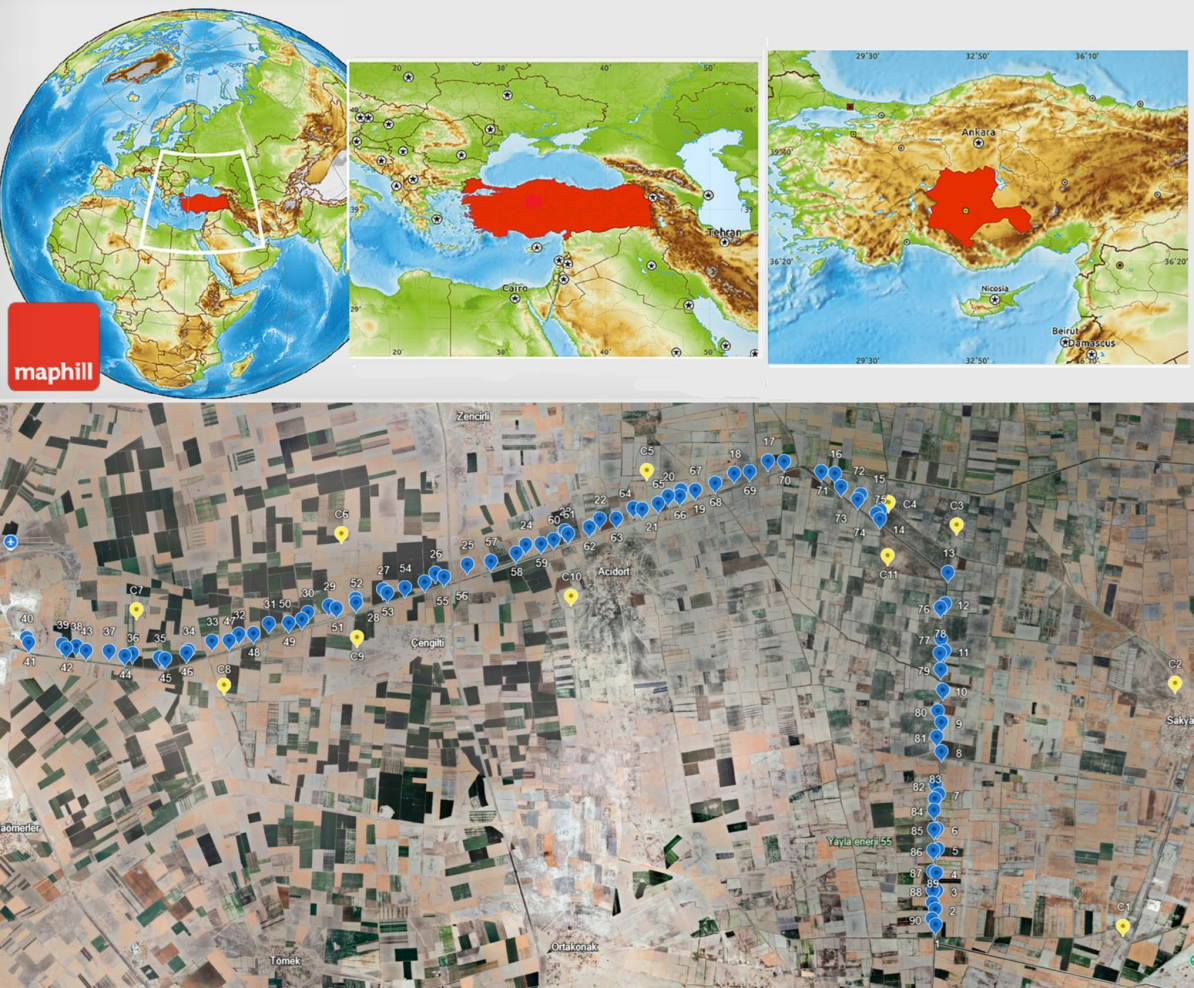
Soil sampling points along the Konya main drainage channel (C1-C11: Control soil sampling points, not irrigated with wastewater, S1-S90: TWW irrigated soil sampling points)

Soil samples were collected from agricultural lands irrigated with Konya WWTP effluents (so called TWW irrigated) along the main drainage channel. Sampling points are marked on Fig. 1. Grab sampling methodology was used to collect soil samples for soil chemical analysis and MPs testing. Soil samples were taken from 90 sampling points at soil depths of 0–10 cm and 10–20 cm by using a stainless steel shovel. Control samples were also taken from 11 non-agricultural locations at the same depths which are distant from the main drainage channel and not irrigated with wastewater. The purpose of collecting control soil samples was to determine the amount of background MPs formed and transported to agricultural soils through different pathways, such as atmospheric wet and dry deposition, and other sources, unrelated to TWW irrigation. There are no additional sources of MP formation, such as highways, plastic recycling facilities, or factories, along the main drainage channel. Even though there was no soil mulching application in the study area, it must be kept in mind that fertilizers may be an additional source of MPs. Separate sampling containers were used for MPs and physicochemical analysis. The samples taken for MPs analysis were wrapped in aluminum foil and placed inside a cotton cloth bag. Soil samples transported to the laboratory were stored in a refrigerator at 4 °C until the analyses were performed.

### Microplastic analysis in soil

#### Preparation of soil samples for analysis

Soil samples were dried at laboratory room temperature before analysis (Li et al. 2020). Agglomerated soil samples were lightly crushed. Visible non-MP impurities such as weeds and plant roots were manually removed. Dried soil samples were sieved through a stainless steel sieve with a pore size of 5 mm (Cao et al. 2021).

#### Separation of microplastics from soil

Density separation was used to isolate MPs from the soil matrix. For this, 100 mL of zinc chloride (ZnCl_2_) was added to 50 g of soil sample, followed by ultrasonic extraction at 35 kHz frequency, for 15 min, at 25 °C. After that, the mixture was stirred at 600 rpm for 30 min. The mixture was left in a fume hood for 24 h for phase separation, with the beakers’ mouths covered with aluminum foil, before proceeding to the next step (Yang et al. 2021).

#### Removal of organic matter

A wet peroxide oxidation procedure was conducted to remove natural organic matter from the MPs. This process followed the method defined by the National Oceanic and Atmospheric Administration (NOAA) (Masura et al. 2015). After the density separation step, the upper phase was filtered through a 20 µm sieve, and the residue remaining on the sieve was washed several times with pure water. The residue on the sieve was transferred to a beaker, and 20 mL of % 30 hydrogen peroxide (H_2_O_2_) and 20 mL of 0.05 M Fe (II) solution were added. The beakers were sealed with aluminum foil and left in a fume hood for 24 h for complete oxidation. The oxidized mixture was filtered through a Millipore brand square filter with a pore size of 0.45 µm. The filter was placed in a glass petri dish and kept in a desiccator until it dried. During the MPs analyses, each filter square was examined under a microscope to count the number, color, and shape of MPs, and the total count was used to determine the MPs concentration in the sample.

#### Identification and characterization of microplastics

Visual examination of the filtered MPs was conducted using a microscope (Nikon Eclipse) with a camera (Nikon DS-L4 and NIS-Elements software) to observe the morphological characteristics of the MPs. The counted MPs were visually classified into fibers, foam, films, pellets, and fragments. MPs were grouped according to their colors: transparent, white, blue, black, green, yellow, brown, pink, and gray. The MPs quantity was expressed as the number of MPs per kilogram of dry soil, denoted as MP/kg.

The characterization of the visually identified MPs was carried out using Fourier Transform Infrared Spectroscopy (FTIR) at wavenumbers from 400 to 4000 cm^−1^, and spectral resolution 4.00 cm^−1^. The polymer types of the MPs were determined by analyzing the obtained FTIR spectra using the OMNIC Specta software, a readily available library (Thermo Fisher Scientific Nicolet iS20). The software includes multi-component search capabilities and 9,000 library spectra.

### Physicochemical analysis of soil

Soil samples were sieved through a 2 mm sieve before use (ISO11464 2006). The clay/silt/sand content of the soil samples was determined using the Bouyoucos hydrometer method. A 50 g sample and 2 g of sodium hexametaphosphate were placed into a beaker; followed by approximately 600 mL of distilled water; and mixed for 5 min with a mixer. The soil suspension was transferred to a graduated cylinder and filled with distilled water up to the 1130 mL mark as the hydrometer was placed inside. The hydrometer was then removed, and the cylinder was inverted several times to ensure complete mixing of the suspension. The first hydrometer and temperature readings were taken after 40 s, and the second readings were taken 2 h later. Adjustments were made for the moisture content of the soil when determining the mass of the samples. The first hydrometer reading provided the silt and clay mass, while the second reading indicated the clay mass. After calculations, the soil texture was determined using a texture triangle (Bouyoucos Hydrometer Method, 1962).

As reported by Richard (1954), the soil sample (100 g) was saturated with distilled water according to specific procedures, and the water content of the soil was determined by measuring its percentage. The pH of the soil samples was measured in soil saturated with water using a glass electrode pH meter (Jackson 1962).

The organic matter content (%) in the soil samples was determined using the modified Walkley–Black method, with 1 g of soil (Nelson and Sommers 1982). Total nitrogen (T-N) in soil samples was determined (%) using the Kjeldahl method with 1 g of soil and a Velp brand nitrogen determination device (pr EN 16169 2012). Plant available phosphorus content (mg/kg) in alkaline and neutral soils was determined using the Olsen method (Olsen et al. 1954).

The relationship between the physicochemical properties of the soil and the amount of microplastics detected was evaluated using correlation and the significance level (Cao et al. 2021). This was carried out using Microsoft Excel and IBM SPSS 22.0. The significance level for the analyses was set at 5%.

### Method validation

Soil samples were placed in glass containers, and plastic materials were avoided during the analyses. To prevent contamination from the air, the samples were covered with aluminum foil. To prevent MPs contamination from clothing fibers and gloves, researchers wore cotton laboratory coats and latex gloves, and wore masks during the analyses. The experiments were conducted in duplicate, and blank analyses were performed simultaneously. Blank analyses were conducted to check for MPs contamination during the analytical steps. For this purpose, blank analyses were performed in parallel with the MPs analysis method (density separation, organic matter removal, filtration, etc.) using soil samples without MPs. No MPs were detected in the blank samples. Deionized water was used throughout all stages of the analyses.

MPs method analyses recovery studies were conducted using a modified method from Rodrigues et al. (2018). For method validation, six common polymer types from everyday life were selected: milk bottles (HDPE), polypropylene containers (PP), CD cases (PS), water pipes (PVC), water bottles (PET), and other bottle caps (LDPE). These samples were grounded to sizes smaller than 5 mm. Since particles smaller than 5 mm are defined as MPs, the ground particles were sieved through a 5 mm sieve, and approximately 0.05 g of each sample was added to the mixture. The number of particles varies according to the material density. A total of 374 MPs (49 from LDPE, 52 from HDPE, 40 from PP, 36 from PS, 47 from PVC, and 150 from PET) were spiked into 20 g of soil samples. Following the MPs analysis method, the recovery rates were calculated, and recovery efficiencies were found to be 90–100% by weight and 94–104% by particle count, confirming the method’s accuracy.

### Risk Assessment

For the estimation of the degree of MPs contamination of the soil along the Konya main drainage channel, contamination factor (CF), pollution load index (PLI), and the polymer risk index (H) were calculated. The CF value for determining the risk level of MPs pollution was calculated using Eq. (1) (Yang et al. 2024). Here, CF_i_ is the contamination factor of MPs, C_i_ is the detected amount of MPs, and C_oi_ is the background value of MPs. The C_oi_ can be taken as the lowest detected MP value. In this study, the lowest MP values detected in control soils taken from a depth of 0–10 cm and 10–20 cm were 100 MP/kg, and 80 MP/kg, respectively. CF < 1 indicate low pollution, 1 ≤ CF < 3 indicates moderate pollution, 3 ≤ CF < 6 indicates significant pollution, and CF ≥ 6 indicates very high pollution (Hakanson 1980; Hossain et al. 2024).

1 $$C{F}_{i}=\frac{{C}_{i}}{Coi}$$

The PLI for each sampling point and entire sampling area were calculated using Eq. (2) and Eq. (3), respectively. Here, PLI represents the MPs load index at a sampling point, and PLI_zone_ refers to the MPs pollution load index for the sampled area. If PLI < 10, the study area is classified as hazard category I; 10 < PLI < 20 indicates hazard category II; 20 < PLI < 30 represents hazard category III; and PLI > 30 indicates hazard category IV (Hossain et al. 2024; Ranjani et al. 2021).

2 $$PLI=\sqrt{C{F}_{i}}$$

3 $$PL{I}_{zone}=\sqrt[n]{PL{I}_{1}\times PL{I}_{2}\times \cdots \times PL{I}_{n}}$$

The H value was used to assess the potential ecological risk of different polymer types and was calculated using Eq. (4). Here, H represents the polymer risk index, P_n_ is the polymer percentage of MPs at each sample point, and Sn is the risk score for the polymer. The Sn values were taken as follows: 11 for PE and LDPE, 1 for PP, 1177 for PC, and 50 for PA (Lithner et al. 2011; Yang et al. 2024). For polymer risk classification, H < 10 indicates risk level I; 10 ≤ H < 100 indicates risk level II; 100 ≤ H < 1000 indicates risk level III; and 1000 ≤ H < 10,000 indicates risk level IV (Chen et al. 2022b; Yang et al. 2024).

4 $$H=\sum {P}_{n}\times {S}_{n}$$

## Results and discussion

### Abundance of the microplastics

**Fig. 2:**
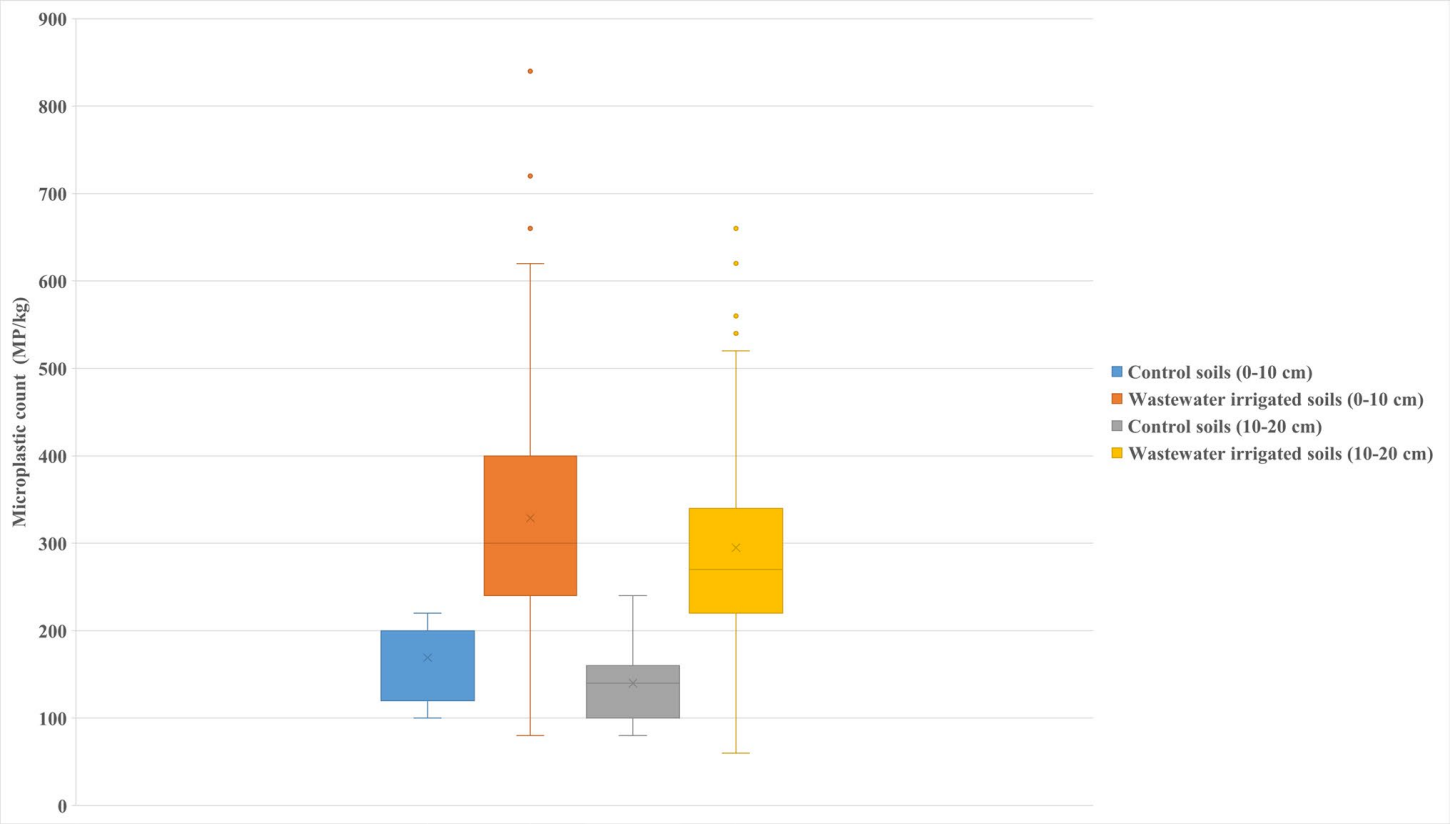
MP count at 0–10 cm and 10–20 cm soil depths (MP/kg)

Our findings revealed that TWW irrigation results in MPs contamination of soil along the Konya main drainage channel. MPs accumulated in the non-agricultural control soil and TWW irrigated soil at depths of 0–10 cm and 10–20 cm were analyzed for their MP count. The statistical values of MPs determined are provided in Table 1. MP count in sampling points is also given in Fig. 2. The average MP count in control soil samples was 169 ± 46.8 MP/kg, whereas it was 329 ± 139.5 MP/kg in soil samples irrigated with TWW at a depth of 0–10 cm. Similarly, the average MP count in control soil samples at a depth of 10–20 cm was 140 ± 44.7 MP/kg, compared to 295 ± 115.4 MP/kg in TWW irrigated soil samples at the same depth. The ANOVA test results (*P* < 0.0001) show a significant difference in MP counts between control and TWW irrigated soils at both depths. These findings suggest that TWW irrigation contributes to an increase in MP abundance in soil. Additionally, the P value (*P* < 0.05) indicates a significant difference in MP counts between the 0–10 cm and 10–20 cm depths, suggesting that MPs are primarily deposited on the soil surface.

**Table 1:** Statistical evaluation of MP count for control and TWW irrigated soil samples

Sample type	Soil depth (cm)	Number of samples	MP count			Standard deviation	*F*	*P*
			min	mean	max			
Control soil TWW irrigated soil	0–10	11 90	100 100	169 329	220 840	46.8 139.5	110.81	< 0.0001
Control soil TWW irrigated soil	10–20	11 90	80 80	140 295	240 660	44.7 115.4	167.40	< 0.0001
Control Control	0–10 10–20	11 11	100 80	169 140	220 240	46.8 44.7	2.96	0.1163
TWW irrigated soil TWW irrigated soil	0–10 10–20	90 90	100 80	329 295	840 660	139.5 115.4	6.27	0.0141

The MP count in TWW irrigated soil samples reported in the literature is presented in Table 2. Only a few studies, focusing on the effect of TWW irrigation on MPs accumulation in soil, have been recently published. The methodology of these research studies varies significantly in terms of MPs’ sizes, sampling depths, wastewater irrigation periods, and analysis methods. Salehi et al. (2023) used a methodology comparable to ours in terms of the method of analysis and reported comparable results for MP count in TWW irrigated soil samples. ZnCl_2_ density extraction followed by H_2_O_2_ digestion was used for MPs separation from soil samples. Soil samples were taken from a 15 cm depth; and MPs (100 nm—5 mm) and mesoplastics (5 mm—20 mm) were identified. MPs content of soil samples irrigated with groundwater, surface water, and TWW, were compared with non-agricultural control soil samples. The highest MP count was 1123 ± 553 MPs/kg for surface water irrigated soil samples. 327 ± 204 MPs/kg were identified in soil samples irrigated with treated wastewater, while there were 41.7 ± 6 MPs/kg in the non-agricultural control site.

**Table 2:** MPs in TWW irrigated terresterial areas

Country	Region	Land type/crop/soil depth	MP count (MPs/kg)	MP size	MP shape	MP polymer type	Reference
Iran	Varamin and Ray Counties	Farmlands/vegetables and cereals/15 cm	769 ± 407(Irrigated with groundwater) 1123 ± 553 (Irrigated with surface water) 327 ± 204 (Irrigated with TWW) 41.7 ± 6 (Control site, uncultivated)	0.037–5 mm	Fibers, fragments, films, foams	PP, PE, PA, polyester (PES), PET, polyurethane (PU), PS, PVC, PC	Salehi et al. (2023)
Tunisia	Ourdanin region	Agricultural soil/20 cm	338.6 ± 30.40 (Soil irrigated with TWW for 5 years) 555.6 ± 30.85 (Soil irrigated with TWW for 16 years) 861.4 ± 8.79 (Soil irrigated with TWW for 24 years) 13.8 ± 1.92 (Control site)	> 3 μm 3–1.22 μm < 1.22 μm	-	PE, PP, HDPE, LDPE, PS, PET	Hattab et al. (2024)
India	Coimbatore	Agricultural soil/0–20 cm	20–84	0.1–5 mm	Fibers, fragments, films, foams, and pellets	PE, PP, PS, PET, PVC	Karthick and Siddhuraju (2024)

Hattab et al. (2024) investigated the impact of MPs accumulation in soil irrigated with TWW over extended periods (5 years, 16 years and 24 years). 338.6 ± 30.40 MPs/kg for 5 years irrigation, 555.6 ± 30.85 MPs/kg for 16 years irrigation, and 861.4 ± 8.79 MPs/kg for 24 years irrigation, were reported, while 13.8 ± 1.92 MPs/kg was identified for the control site. Lower abundance of MPs in both wastewater and well water irrigated soil samples was reported by Karthick and Siddhuraju (2024). They used NaCl density extraction followed by H_2_O_2_ + H_2_SO_4_ digestion for determination of MPs < 5 mm. Soil samples were collected from four agricultural lands irrigated with wastewater and one agricultural land irrigated with well water in Coimbatore, India. While 20–84 MPs/kg was determined in wastewater irrigated soil samples, 20 MPs/kg was determined in well water irrigated soil samples.

The input of MPs into WWTPs, the efficiency of MPs removal by WWTPs, the duration of treated or non-treated wastewater irrigation, and various other factors all influence the abundance of MPs in soil. There is also a correlation between soil physicochemical properties and the abundance of MPs in soil, which was one of the subjects of this study and is discussed under Sect."Risk assessment of microplastics"subtitle. While large quantities of MPs can be removed in urban WWTPs, inputs from stormwater runoff and industrial discharges may lead to increased plastic contamination (Carr et al. 2016). Even after successful secondary treatment, a significant number of MPs can still be present in the effluent (Raju et al. 2018).

### Morphological characterization of microplastics

**Fig. 3:**
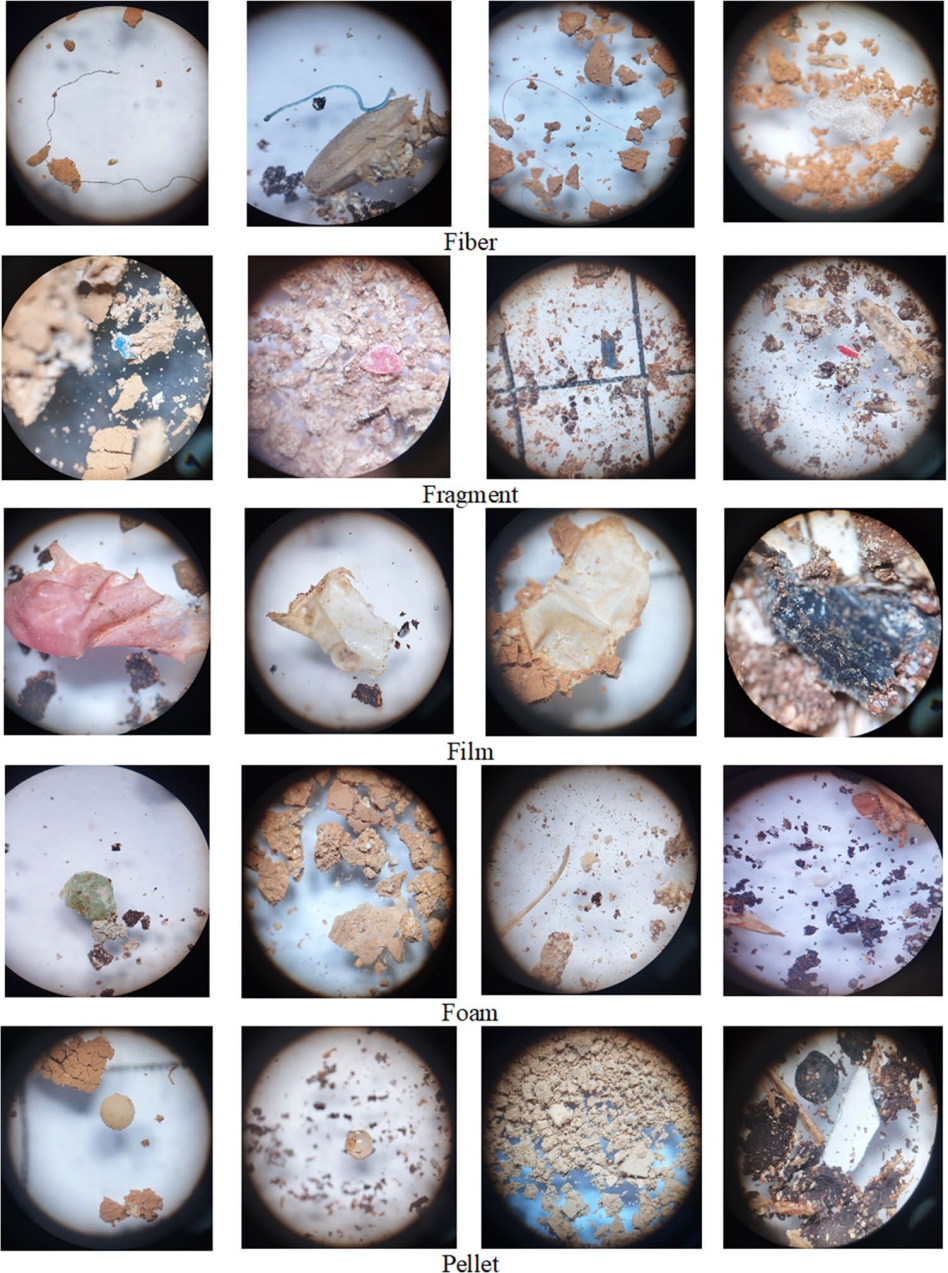
Images of some MPs found in the soil samples taken from the TWW irrigated field

**Fig. 4:**
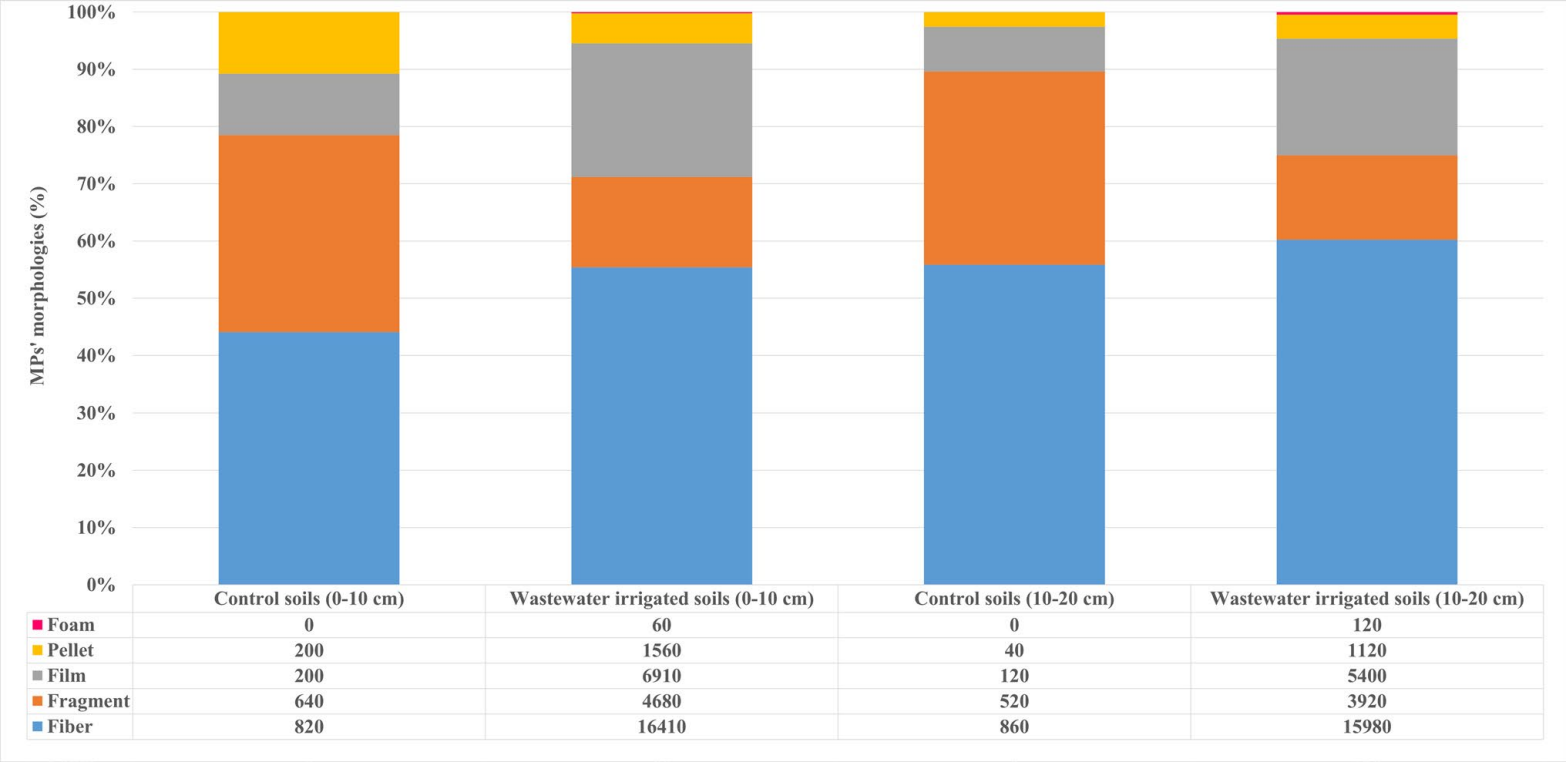
Total accounts and morphologies percents of MPs in control and TWW irrigated soils

Five morphological types of MPs were identified in soil samples: film, fragment, pellet, foam, and fiber. Images of some of the MPs visually identified in the soil samples are shown in Fig. 3, and the percentages of MPs’ morphologies in both controls, and TWW irrigated soil samples are provided in Fig. 4. In the 0–10 cm soil samples, the most common MP shapes were fibers (56%) > films (23%) > fragments (16%) > pellets (5%) > foam (0.2%). While fiber, fragment, film, and pellet-shaped MPs were found in both control and TWW irrigated soils, foam-shaped MPs were only detected in TWW irrigated soils. A significant difference (*P* < 0.0001) was observed in the number of fiber and film-shaped MPs between the control and TWW irrigated soils at 0–10 cm depth.

While no studies have been conducted on the shapes of MPs in the effluent of Konya Urban WWTP, it is well established that synthetic textile products are significant sources of fibers in wastewater. Specifically, MP fibers shed from synthetic fabrics during washing in washing machines are commonly found in wastewater (Ziajahromi et al. 2017). Film-like MPs in wastewater typically originate from the use of plastic films and thin plastic materials across various industries. Single-use plastic packaging, commonly used in the food and beverage sector (e.g., plastic bags, bottles, cling film), can release MPs into wastewater. These films may result from the degradation or fragmentation of plastic packaging. Additionally, synthetic textile products (e.g., polyester, nylon) can release thin plastic particles, such as films and fragments, during washing. Fragments and pellets are also commonly used in cosmetic microbeads (Mason et al. 2016).

Mason et al. (2016) conducted research on plastics content of 17 WWTPs effluents throughout the United States. Findings demonstrated that between 50,000 and 15,000,000 particles per day could be released from each plant. The most common MP shapes were fibers (59%) > fragments (33%) > films (5%) > foams (2%) > pellets (1%). Salehi et al. (2023) and Karthick and Siddhuraju (2024) also determined fibers, fragments, films, and foams in soil samples taken from TWW irrigated lands.

### Colors of microplastics

**Fig. 5:**
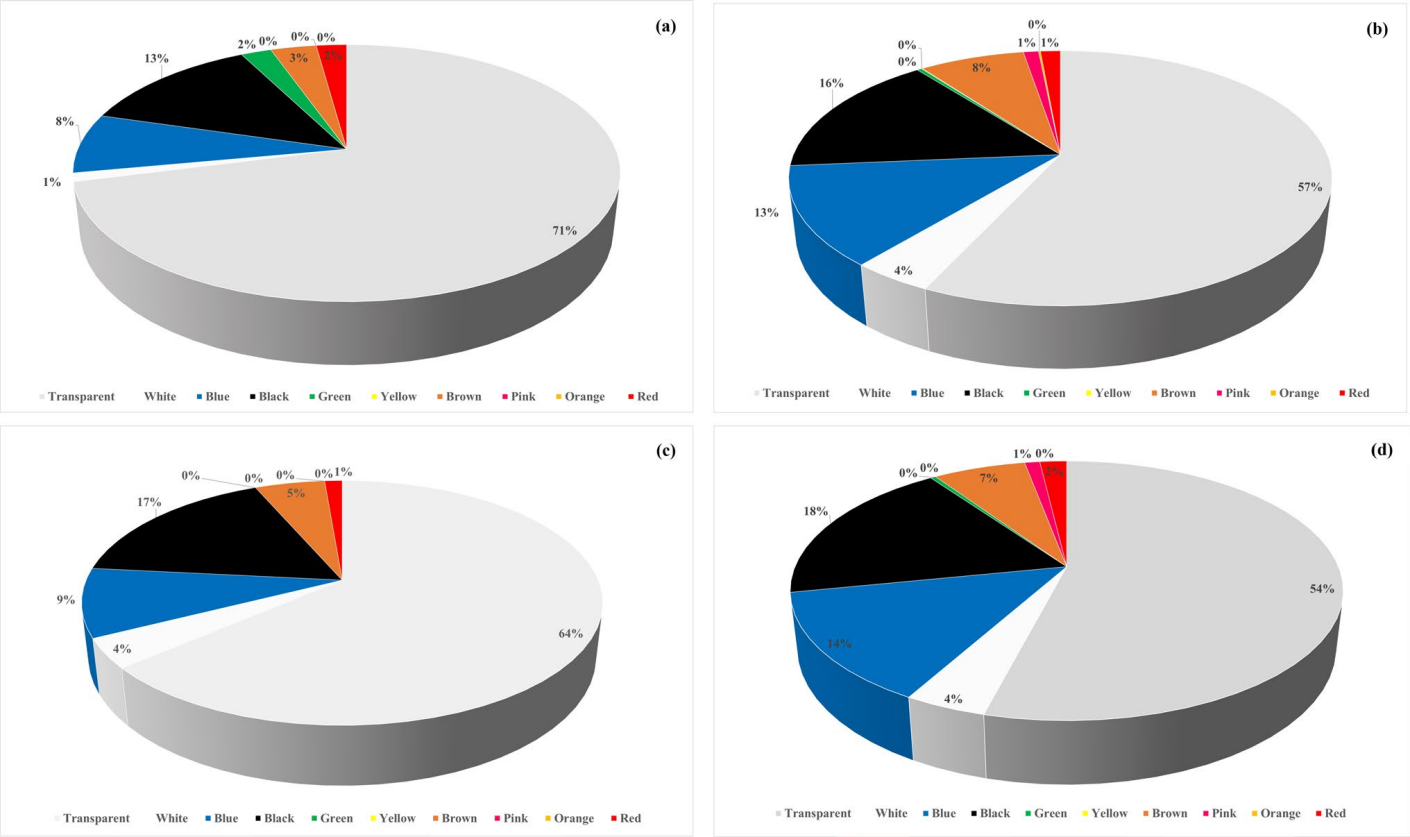
Colors of MPs determined in (**a**) control soil (0–10 cm), (**b**) wastewater irrigated soil (0–10 cm), (**c**) control soil (10–20 cm), (**d**) wastewater irrigated soil (10–20 cm)

The color distribution ratios of the detected MPs are shown in Fig. 5. Transparent MPs were predominantly detected in both control (0–10 cm; 71%, 10–20 cm; 64%) and TWW irrigated soils (0–10 cm; 57%, 10–20 cm; 54%). Comparing control soils with TWW irrigated soils, TWW irrigation increased the proportion of different types of dark-colored MPs. Transparent, white, blue, black, green, brown, and red MPs were detected in control soils at a depth of 0–10 cm, while, in soils irrigated with TWW, in addition to these colors, yellow, pink, and orange MPs were also found. In control soils at a depth of 10–20 cm, transparent, white, blue, black, brown, and red MPs were identified, while in wastewater-irrigated soils, green and pink MPs were additionally detected. Black and transparent have been the most abundant colors of MPs found in soil in previous studies. Zhang et al. (2024) reported 82.2% black MPs, followed by transparent MPs (9.6%), in soils sampled in Southwest China. Karthick and Siddhuraju (2024) found that wastewater irrigated lands in Coimbatore, India, contained 57% black MPs, followed by white (18%), blue (11%), and transparent (10%). White MPs (30.0%), followed by transparent MPs (23.5%), and black MPs (20.0%) were reported by Salehi et al. (2023). Black MPs can originate from packaging made of black plastic, the wear of vehicle tires, the washing of synthetic textile products. Transparent and white MPs generally originate from single-use plastic products and agricultural greenhouse and mulching covers in the field, while blue MPs are often attributed to the widespread use of blue in the textile industry. Additionally, transparent and white MPs may form from colorless plastic products, or the colors of waste plastics in the environment may fade due to factors such as pH, sunlight, and temperature.

### Polymer types of microplastics

**Fig. 6:**
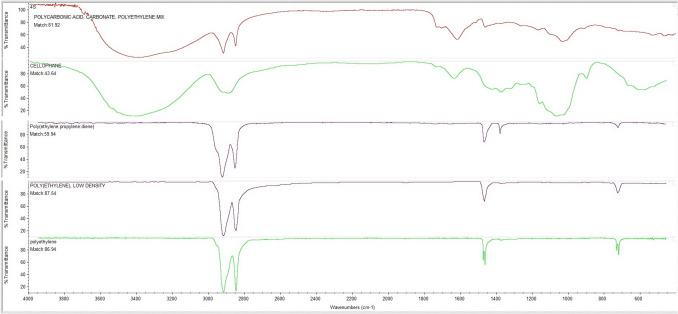
FTIR spectra of the MPs

**Fig. 7:**
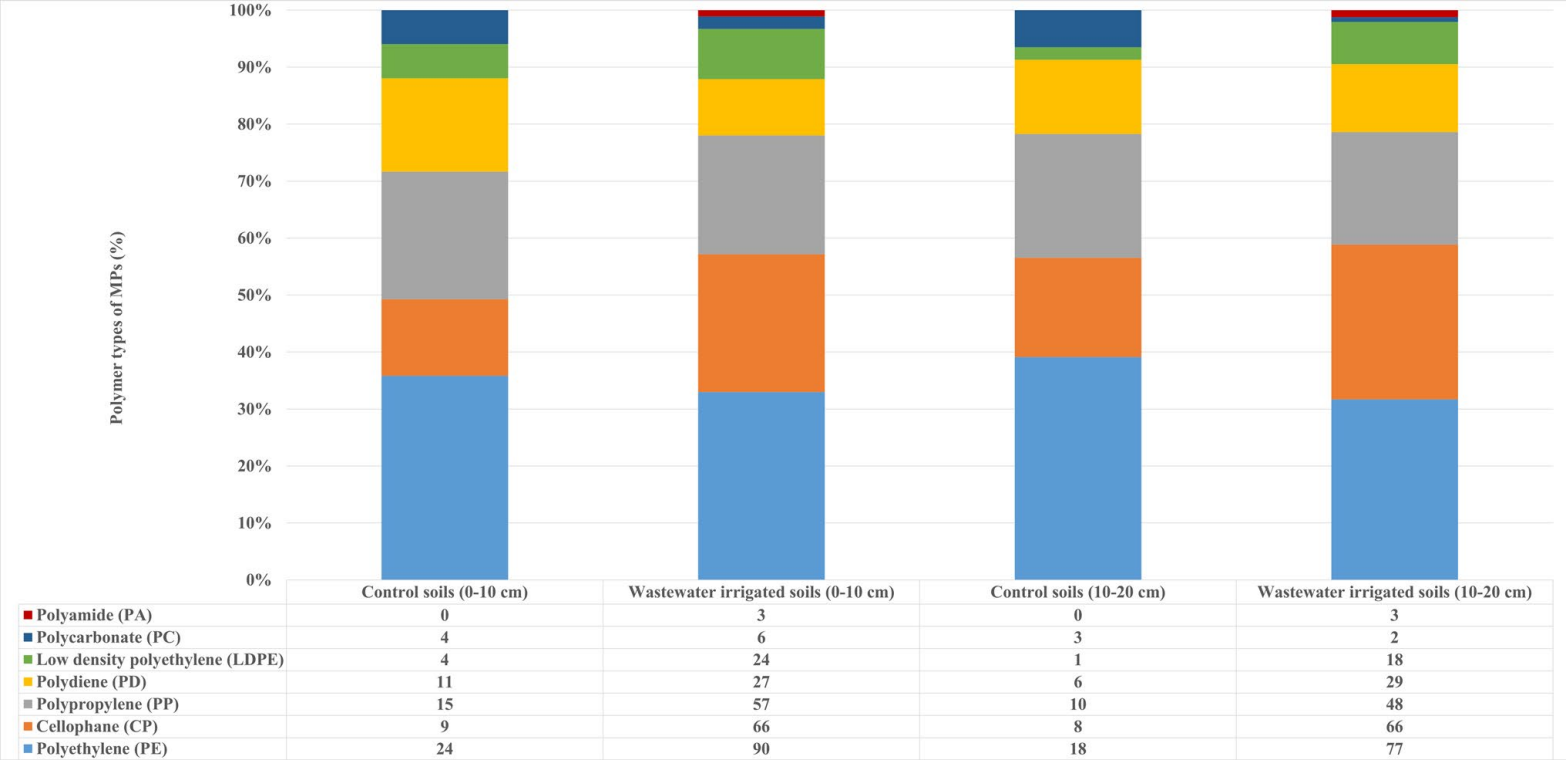
Percents of polymer types of MPs in the control and TWW irrigated soils

The polymer types of MPs were identified by comparing the obtained FTIR peaks with the readily available OMNIC Specta software library. The peaks were also compared with the absorption band data reported for each plastic polymer in the literature. The FTIR spectrums are shown in Fig. 6. PE, cellophane (CP), PP, polydiene (PD), LDPE, and PC have been detected in TWW irrigated soil samples and control soil samples, according to FTIR analysis. In TWW irrigated soils, PA was also detected, unlike in the control soils. In both TWW irrigated and control soils, PE, CP, and PP polymers were the most abundant polymer types. In control soils sampled from a depth of 0–10 cm, PE was detected at 36%, PP at 27%, PD at 16%, CP at 14%, and LDPE and PC at 6%. In TWW irrigated soils, PE was detected at 33%, CP at 24%, PP at 23%, PD at 10%, LDPE at 9%, PC at 2%, and PA at 1% (Fig. 7). The polymer types and their proportions detected in soils sampled from a depth of 0–20 cm showed a variation ranging from 1 to 3%.

The PE and PP, which are predominantly found, are the most commonly used polymers in plastic production. PE is frequently utilized due to its low cost, ease of shaping, mechanical qualities, and versatility (Karthick and Siddhuraju 2024). PE is commonly used as shopping bags, beverage bottles, detergent containers, agricultural mulch film, wastewater pipes, and irrigation systems (Islam and Cheng 2024). Cost-effectiveness, resistance to breakdown, and mechanical properties make PP one of the most used plastic polymers. PP is commonly used in the production of food packages, snack products, automotive parts, and pipes (Islam and Cheng 2024). The types of polymers in MPs in soil depend on the composition of WWTP’s effluents. Majewsky et al. (2016) determined 80–260 mg/m^3^ of PE and PP in domestic wastewater.

Salehi et al. (2023) identified 33.5% PP and 18.0% PE in plastic samples taken from farmlands irrigated with wastewater. The dominant MPs in the agricultural lands of Xinjiang Province were identified as PE, which was attributed to plastic mulching (Huang et al. 2020). In Swiss floodplain soils, PE was the most common polymer, followed by PA, PS, and PVC (Scheurer and Bigalke 2018). In agricultural soils of Hangzhou Bay, PE accounted for 75% of the total polymers, followed by PP (18%) and PA (5%) (Zhou et al. 2020).

### The relationship between soil physicochemical properties and microplastic abundance

#### Soil texture

The clay/silt/sand content of the soil samples, determined using the Bouyoucos Hydrometer method, showed that the control soils and TWW irrigated soils were characterized as heavy-textured silty clay and clay soils or medium-heavy textured clay loam soils. It has been reported that the addition of MPs to soils with high clay content has a stronger impact on soil properties compared to non-clayey soils (Mbachu et al. 2021). In clay-loam soils, soil aggregate formation increased with PES-MP application, resulting in larger water-stable aggregates (Zhang et al. 2019), while in sandy loam, the formation of water-stable aggregates decreased (de Souza Machado et al. 2018).

#### Soil water saturation

The water saturation value of the soil reflects its porosity and is closely related to the soil’s permeability and water content, making it an important parameter in agricultural production. It also affects the transport of MPs in the soil. The required porosity value for plant growth and development should be between 50–60%. Porosity values are classified as low (< 50%), medium (50–60%), and very high (> 60%) (Zhang et al. 2024). The water saturation values of the control soils ranged from 49.5% to 72.6%, while those of the TWW irrigated soils ranged from 49.5% to 103.4%, at 0–10 cm depth. For the control soils, the water saturation values ranged from 57.2% to 79.3%, while those of the TWW irrigated soils ranged from 47.4% to 85.8% at 10–20 cm depth. It was observed that the water saturation values of the TWW irrigated soils were higher than those of the control soils. Based on the *F* and *P* values obtained from the ANOVA test, a significant difference was observed between the water saturation values of the control and TWW irrigated soils at both 0–10 cm depth (*F*: 24.37, *P*-value: 0.0000033) and 10–20 cm depth (*F*: 4.924, *P*-value: 0.028). A significant MP count change was not observed in the control soils. Similarly, a consistent change in MP count and water saturation of TWW irrigated soil was not evident. The relationship between soil water saturation and the MP count detected in the soil was assessed using the Pearson correlation coefficient, but no significant correlation was found. Zhang et al. (2024) reported a slight decrease in MP count with the increase in soil total porosity, which was attributed to facilitated immigration of MPs into deeper soil. According to Zhang et al. (2022), soils affected by MPs tend to be more porous and have a higher water saturation capacity; however, it was found that this did not improve soil stability or increase soil microbial diversity. These findings suggest that MPs occupy physical space but do not integrate into the soil's biophysical matrix.

#### Soil pH

Another effect of MPs on soil properties is their ability to alter soil pH (Palansooriya et al. 2022). MPs can interact with other substances in the soil and change the pH, making it more acidic or alkaline (Khalid et al. 2021). The pH values of the control soil ranged from 7.05 to 7.93, while the pH values of the TWW irrigated soil ranged from 6.87 to 8.06, at 0–10 cm depth. The pH values ranged from 7.54 to 8.57 for the control soils and from 6.80 to 8.12 for the TWW irrigated soil at 10–20 cm depth. At both depths, the pH range in the control soils was wider, whereas the pH variation in the TWW irrigated soils occurred within a narrower range. According to the *F* and *P* values obtained from the ANOVA test, no significant difference was observed between the pH values of the control and TWW irrigated soils at 0–10 cm depth (*F*: 0.238, *P*-value: 0.626). However, a significant difference was observed between the pH values of the control and TWW irrigated soils at 10–20 cm depth (*F*: 18.31, *P*-value: 0.000043). As the soil depth increased, the pH values in both the control and TWW irrigated soils increased. The relationship between soil pH and the MP count detected in the soil was evaluated using the Pearson correlation coefficient, but no clear correlation was found. Even though no significant effect of MPs count on soil pH was determined in this research, there are many studies revealing the effect of MPs on soil pH. Wang et al. (2022) have shown that MPs can lead to the release of potentially toxic chemicals and increase soil acidity due to the adsorption of protons. Qi et al. (2020) reported that exposure of soil to a MP mixture increases the soil pH. In contrast, after 30 days of exposure to high-density polyethylene, the soil pH decreased, which was attributed to the release of lactic acid from aliphatic polyesters (Bandow et al. 2017; Boots et al. 2019).

#### Soil organic matter

MPs can also affect the soil organic matter content. Since organic matter provides energy and nutrients to soil microorganisms, it is important for soil health and nutrient cycling (Liang et al. 2021; Shen et al. 2022). When present in the soil, MPs can interact with organic matter and reduce its availability to soil microorganisms, thereby affecting nutrient cycling and overall soil health (Lan et al. 2022). The organic matter values of control soils ranged from 1.83% to 6.57% at 0–10 cm depth, while the organic matter values of TWW irrigated soil ranged from 1.59% to 5.83%. The organic matter values of control soils at 10–20 cm depth range from 1.88% to 6.26% while TWW irrigated soil range from 1.36% to 4.77%. In TWW irrigated soils at 0–10 cm depth, soils with low levels (1–2%) of organic matter contained 3,540 MP/kg, moderate levels (2–3%) contained 12,700 MP/kg, and high levels (3–5%) contained 12,960 MP/kg. Similarly, a total of 2,540 MP/kg, 14,660 MP/kg, and 9,360 MP/kg were found for low levels, moderate levels, and high levels of organic matter at 0–20 cm depth. As the amount of organic matter in the soil increased, the number of MPs detected also increased. This can be explained because the increase in organic matter stimulates microbial activity, thereby enhancing the degradation of MPs.

A positive correlation between organic matter and MP content of soil has been observed in some studies, and this is likely explained by the fact that the increase in organic matter stimulates microbial activity, thereby enhancing the degradation of larger plastics to MPs, (Zhang et al. 2024; Liu et al. 2017). The organic matter content of the soil is controlled by soil microorganisms through decomposition (Kang et al. 2021). Therefore, when the soil is contaminated with MPs, the abundance of microorganisms in the soil will decrease, which means that the decomposition of organic matter by microorganisms will also decrease (Chia et al. 2022). However, there is a need for more studies to highlight this fact.

#### Soil total nitrogen

Soil nitrogen (N) is an important plant nutrient. The nitrogen mineralization-immobilization cycle, which is the conversion of organic N to inorganic N carried out by soil microorganisms, together with its reverse process, is thought to have a significant impact on the amount of biologically available N in the soil (Nannipieri and Eldor 2009). The accumulation of MPs in the soil can directly or indirectly affect the functioning of soil ecosystems (Bandow et al. 2017; Luo et al. 2020), which in turn can further influence soil N mineralization and bioavailability. In this study, the total nitrogen values of the control soils ranged from 0.09% to 0.61%, while the values for TWW irrigated soils ranged from 0.04% to 0.68% at 0–10 cm depth. Similarly, total nitrogen values ranged from 0.09% to 0.61%, and 0.04% to 0.59% for control and TWW irrigated soils, respectively, at 10–20 cm depth. No significant difference was observed between the total nitrogen values of the control soils and those of TWW irrigated soils (*F*: 0.004, *P*-value: 0.949 for 0–10 cm; *F*: 1.007, *P*-value: 0.318 for 10–20 cm).

TWW irrigated soils with low nitrogen levels (0.045—0.09%) contained 4,540 MP/kg; adequate nitrogen levels (0.09—0.17%) contained 8,520 MP/kg; high nitrogen levels (0.17–0.32%) contained 5,200 MP/kg; and very high nitrogen levels (> 0.32%) contained 10,720 MP/kg, at 0–10 cm depth. Similarly, 1,660 MP/kg, 7,040 MP/kg, 5,680 MP/kg, and 11,940 MP/kg were determined for low, adequate, high, and very high nitrogen levels, respectively, at 10–20 cm depth. In both soil depths, it was observed that, as the soil nitrogen level increased, the MP count detected in the soil also increased. This can be explained by the increase in microbial activity due to the rise in total nitrogen, thereby enhancing the breakdown of MPs. Some studies conducted in the last decade have shown that soil pollution caused by MPs can lead to an increase in soil nitrogen content (Fei et al. 2020; Zhu et al. 2022). This increase may be related to the rise of *Burkholderiaceae* bacteria associated with soil contamination by MPs (e.g., PVC and PE). These bacteria belong to a family of biological nitrogen-fixing bacteria that can enhance soil nitrogen storage (Fei et al. 2020). Additionally, according to Rong et al. (2021), soil contamination by MPs, alters the nitrogen cycle by increasing the abundance of nitrogenase reductase genes in bacteria involved in the nitrogen cycle. Since the carbon/nitrogen ratio changes due to MPs contamination, the nitrogen cycle is also affected (Khalid et al. 2021).

#### Soil available phosphorus

One of the main beneficial effects of phosphorus on soils is the promotion of growth and crop production (Spohn 2020). The available phosphorus values for plants in control soils ranged from 14.8 to 114 mg/kg at 0–10 cm depth, while the values for TWW irrigated soils ranged from 15.2 to 160 mg/kg. For the control group, the available phosphorus values ranged from 7.25 to 80.0 mg/kg, and for TWW irrigated soils, the values ranged from 5.85 to 125 mg/kg. It was observed that the available phosphorus values for TWW irrigated soils are higher than those of the control soils. According to the ANOVA test, a significant difference was observed between the available phosphorus values in control and TWW irrigated soils (*F*: 12.26, *P*-value: 0.0006 at 0–10 cm depth and *F*: 17.04, *P*-value: 0.00007 at 10–20 cm depth). In soils with moderate levels of phosphorus, a total of 3,740 MP/kg were determined; in soils with high levels of phosphorus, 7,200 MP/kg were determined; and in soils with very high levels of phosphorus, 18,680 MP/kg were determined for TWW irrigated soils, at 0–10 cm depth. In TWW irrigated soils, phosphorus levels of 5,880 MP/kg were found in soils with moderate phosphorus levels; 6,380 MP/kg in soils with high phosphorus levels; and 13,560 MP/kg in soils with very high phosphorus levels, at a soil depth of 10–20 cm. As the amount of available phosphorus in the soil increased, the number of detected MPs also increased. This can be explained by an increase in available phosphorus in the soil, which encourages microbial activity and, in turn, may increase the breakdown of MPs. When the relationship between available phosphorus in the soil and the MP count detected in the soil was evaluated using the Pearson correlation coefficient, it was found that there was no clear correlation. The contamination of soil with 31% PE, 79% PP, and 1.0% PVC MPs has led to a reduction in the phosphorus content of the soil (Yan et al. 2020). The decrease in soil phosphorus following MPs contamination may be related to changes in the size of soil aggregates, which could lead to a decrease in soil bulk density (Jiang et al. 2017).

### Risk assessment of microplastics

**Fig. 8:**
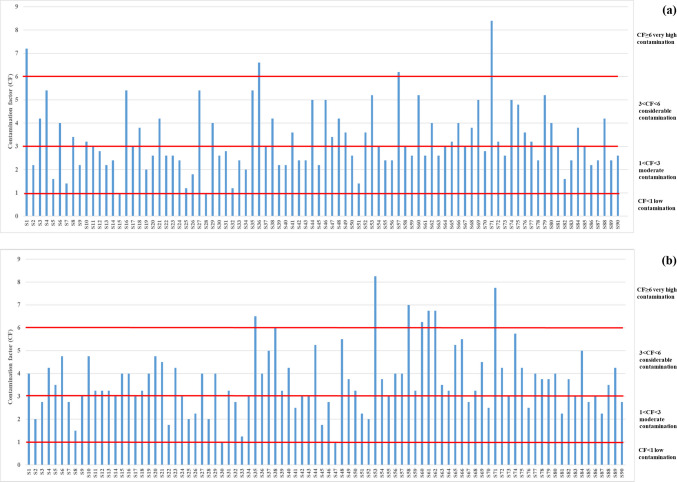
Contamination factors (CF) of (**a**) wastewater irrigated soil (0–10 cm) and (**b**) wastewater irrigated soil (10–20 cm)

The ecological and health risks caused by exposure to MPs are the most studied topics in soil MP research. MPs can directly affect plant growth as well as indirectly influence plants by affecting soil properties or microorganisms. The findings of this study revealed significant contamination of MPs contamination along the Konya main drainage channel, as a result of TWW irrigation. In this study, the risk level of MPs pollution was determined using the Contamination Factor (CF), which depends on the ratio of MP counts in TWW irrigated soil to control soil (Yang et al. 2024). CF values for each sampling point are given in Fig. 8. The highest CF value of 8.40 was determined in the sampling site S71, (0–10 cm). While only 3.3% of the soil samples were exposed to very high contamination (CF ≥ 6 for 3 soil samples), 50% of the soil samples were exposed to considerable contamination (3 < CF < 6 for 45 soil samples) for the 0–10 cm sampling depth. Higher contamination was determined by 10–20 cm depth, while nearly 9% of the soil samples were exposed to very high contamination (CF ≥ 6 for 8 soil samples), 65% of the soil samples were exposed to considerable contamination (3 < CF < 6 for 58 soil samples). The remaining soil samples have all been found to be moderate contamination (1 < CF < 3), while none of the soil samples were found to be of low contamination (CF < 1). Although more MPs were detected at a soil depth of 0–10 cm, more samples with significant levels of MPs were found at a depth of 10–20 cm. This can be explained by the relatively low levels of MPs in the control soils at a depth of 10–20 cm. The area from which the control soils were taken is non-agricultural land the soil has not been disturbed. In these soils, MPs can migrate naturally only to deeper layers. However, in TWW irrigated soils, agricultural activities involve plowing, which transports MPs to the lower soil layers.

**Fig. 9:**
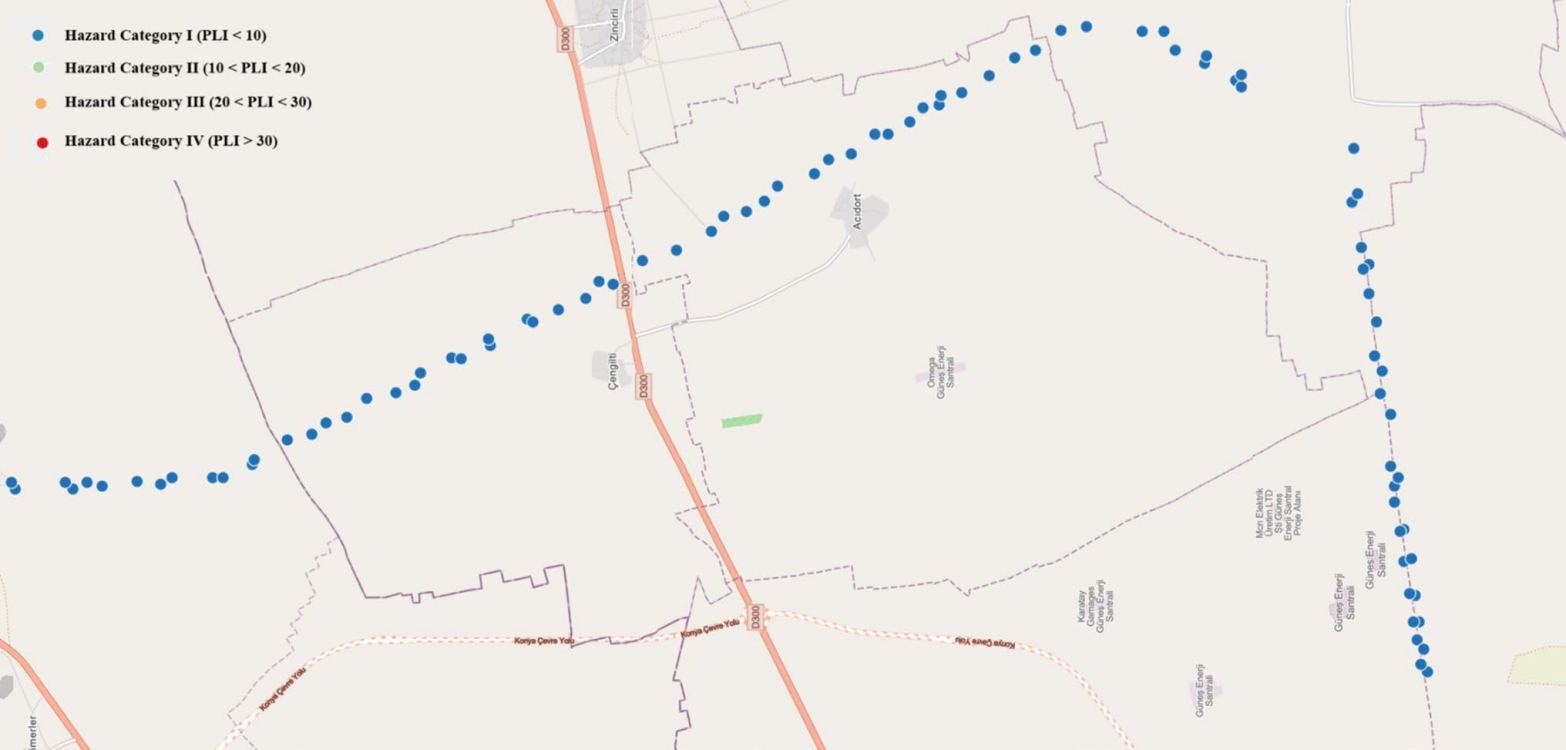
Pollution Load Index (PLI) of wastewater irrigated soil (both for 0–10 cm and 10–20 cm)

Based on the CF values, the Pollution Load Index (PLI) for each soil sampling point and the overall zone (PLI_zone_) were calculated. Results are given in Fig. 9. on the map of the main drainage channel. The marked points represents both 0–10 cm and 10–20 cm soil debths, since all points were in the same Hazard Category I. The highest PLI value was observed at sampling site S71 (2.9) at 0–10 cm depth, followed by S53 (2.87) at 10–20 cm, S1 (2.68) at 0–10 cm, and S36 (2.57) at 10–20 cm depth. The average PLI values for all sampling points were 1.78 ± 0.37 at 0–10 cm depth and 1.88 ± 0.37 at 10–20 cm depth. The PLI_zone_ values were 1.71 at 0–10 cm depth and 1.86 at 10–20 cm depth. These results suggest that the soil along the Konya main drainage channel is contaminated with MPs, as both PLI and PLI_zone_ values exceeded 1. Given that PLI values below 10 indicate Hazard Category I, all sampling points fall into this category (Hossain et al. 2024). PLI values consistent with our findings have been reported by Hossain et al. (2024). The PLI of 1.24 ± 0.13 for soil samples at a dumping site in Bangladesh was determined.

**Fig. 10:**
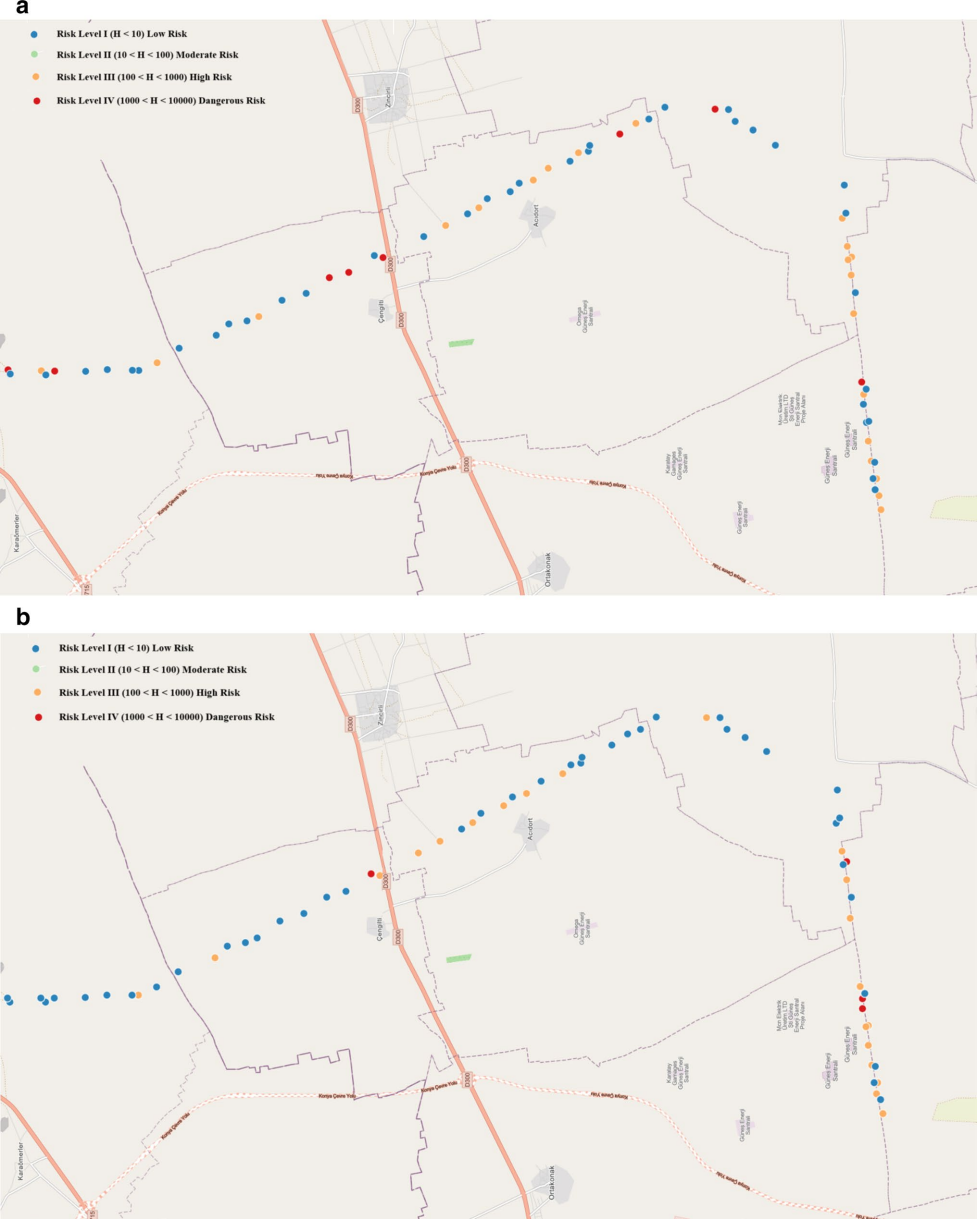
Polymer risk classification (H) of (**a**) wastewater irrigated soil (0–10 cm) and (**b**) wastewater irrigated soil (10–20 cm)

Polymeric Risk Assessment (H) values were determined depending on the polymer percentage of MPs and the risk score of the polymer. Findings are presented in Fig. 10. Eight sampling points at 0–10 cm and four sampling points at 10–20 cm depth exceeded the defined maximum risk level (Risk Level IV; 1000 ≤ H < 10,000), with H values in the range of 10,483 to 17,457, indicating levels beyond this defined risk category. These points are also indicated in red in Fig. 10, highlighting dangerous risk. 67% of the soil samples were under low risk (Risk Level I; H < 10), 23% were under high risk (Risk Level III; 100 ≤ H < 1000), and nearly 9% were under dangerous risk (Risk Level IV; 1000 ≤ H < 10,000) or (H ≥ 10,000) at 0–10 cm depth. Similarly, 72%, 23%, and 4% of the soil samples at 10–20 cm were under low risk (Risk Level I), high risk (Risk Level III), and dangerous risk (Risk Level IV), respectively.

While the area studied falls under Hazard Category I according to the PLI values, some of the sampling points are classified as Risk Level III or Risk Level IV based on the H values. Similar findings were reported by Jia et al. (2024). While PLI values ranged from 1.00 to 2.48, the risk category of the sampling area was determined to be Risk Level II and Risk Level III based on the H values in an agricultural area in Yan’an City, China.

## Conclusions

This study revealed the presence and effects of MPs pollution in TWW irrigated agricultural lands. TWW irrigation increases MPs accumulation in soils, which can adversely affect agricultural ecosystems. A significant increase in MPs was observed in TWW irrigated soils compared to controls. MPs detected include fiber, fragment, film, pellet, and foam, with fiber and film being dominant. The predominant colors were transparent, white, blue, and brown, and PE, CP, and PP polymers were the most common.

Soil texture analysis showed that soils were primarily heavy-textured silty clay, clay, or clay loam. Water saturation values indicated that as saturation increased, MPs accumulation also rose. Higher organic matter, nitrogen, and phosphorus levels also correlated with increased MPs, likely due to enhanced microbial activity.

CF assessment revealed that 47% of 0–10 cm depth soils were moderately polluted, 50% significantly polluted, and 3% heavily polluted with MPs. At 10–20 cm depth, 27% were moderately polluted, 64% significantly polluted, and 9% heavily polluted. PLI assessment classified the soils into danger category I (PLI < 10). According to the H assessment, 23% of the samples from both depths were under high risk, while 9% of the soils from the 0–10 cm depth, and 4% of the soils from the 10–20 cm depth were under dangerous risk.

MPs can persist in soil for decades, accumulate, and impact organisms and biodiversity. They can also transfer pollutants, posing risks to agricultural yield and food security. The potential toxic effects on soil organisms further threaten ecological balance. These findings provide essential data for the sustainable use of TWW in agriculture and the need for regulations addressing MPs pollution and human health.

In conclusion, this study highlights the impact of wastewater irrigation on MP pollution, suggesting the need for further research. It is vital to reassess wastewater management and irrigation strategies to ensure sustainability and reduce environmental risks, alongside standardizing MP analysis in soils.

## Data Availability

Data is available on request from corresponding author.

## References

[CR1] Baeza C, Cifuentes C, González P, Araneda A, Barra R (2020) Experimental exposure of Lumbricus Terrestris to microplastics. Water Air Soil Pollut 231:6. 10.1007/s11270-020-04673-0

[CR2] Bandow N, Will V, Wachtendorf V, Simon FG (2017) Contaminant release from aged microplastic. Environ Chem 14:394–405. 10.1071/en17064

[CR3] Blasing M, Amelung W (2018) Plastics in soil: analytical methods and possible sources. Sci Total Environ 612:422–435. 10.1016/j.scitotenv.2017.08.086 28863373 10.1016/j.scitotenv.2017.08.086

[CR4] Boots B, Russell CW, Green DS (2019) Effects of microplastics in soil ecosystems: above and below ground. Environ Sci Technol 53:11496–11506. 10.1021/acs.est.9b03304 31509704 10.1021/acs.est.9b03304

[CR5] Bosker T, Bouwman LJ, Brun NR, Behrens P, Vijver MG (2019) Microplastics accumulate on pores in seed capsule and delay germination and root growth of the terrestrial vascular plant Lepidium sativum. Chemosphere 226:774–781. 10.1016/j.chemosphere.2019.03.163 30965248 10.1016/j.chemosphere.2019.03.163

[CR6] Bouyoucos GJ (1962) Hydrometer method improved for making particle size analysis of soil. Agron J 54:464–465. 10.2134/agronj1962.00021962005400050028x

[CR7] Cao L, Wu D, Liu P, Hu W, Xu L, Sun Y, Wu Q, Tian K, Huang B, Yoon SJ, Kwon BO, Khim JS (2021) Occurrence, distribution and affecting factors of microplastics in agricultural soils along the lower reaches of Yangtze River, China. Sci Total Environ 794:148694. 10.1016/j.scitotenv.2021.148694 34198075 10.1016/j.scitotenv.2021.148694

[CR8] Carr SA, Liu J, Tesoro AG (2016) Transport and fate of microplastic particles in wastewater treatment plants. Water Res 91:174–182. 10.1016/j.watres.2016.01.002 26795302 10.1016/j.watres.2016.01.002

[CR9] Chen J, Wu J, Sherrell PC, Chen J, Wang H, Zhang W, Yang J (2022a) How to Build a Microplastics-Free Environment: Strategies for Microplastics Degradation and Plastics. Recycling Adv Sci 9:2103764. 10.1002/advs.202103764 10.1002/advs.202103764 PMC8867153 34989178

[CR10] Chen Y, Gao B, Xu D, Sun K, Li Y (2022b) Catchment-wide flooding significantly altered microplastics organization in the hydro-fluctuation belt of the reservoir. Iscience 25:104401. 10.1016/j.isci.2022.104401 35637732 10.1016/j.isci.2022.104401 PMC9142631

[CR11] Chia RW, Lee JY, Jang J, Kim H, Kwon KD (2022) Soil health and microplastics: a review of the impacts of microplastic contamination on soil properties. J Soils Sediments 22:2690–2705. 10.1007/s11368-022-03254-4

[CR12] Corradini F, Meza P, Eguiluz R, Casado F, Huerta-Lwanga E, Geissen V (2019) Evidence of microplastic accumulation in agricultural soils from sewage sludge disposal. Sci Total Environ 671:411–420. 10.1016/j.scitotenv.2019.03.368 30933797 10.1016/j.scitotenv.2019.03.368

[CR13] de Souza Machado AA, Kloas W, Zarfl C, Hempel S, Rillig MC (2018) Microplastics as an emerging threat to terrestrial ecosystems. Glob Chang Biol 24:1405–1416. 10.1111/gcb.14020 29245177 10.1111/gcb.14020 PMC5834940

[CR14] de Souza Machado AA, Lau CW, Kloas W, Bergmann J, Bachelier JB, Faltin E, Becker R, Gorlich AS, Rillig MC (2019) Microplastics can change soil properties and affect plant performance. Environ Sci Technol 53:6044–6052. 10.1021/acs.est.9b01339 31021077 10.1021/acs.est.9b01339

[CR15] EN 16169 (2012) Sludge, treated biowaste and soil - Determination of Kjeldahl nitrogen. Retreived 06.01.2025, https://standards.iteh.ai/catalog/standards/cen

[CR16] Fei Y, Huang S, Zhang H, Tong Y, Wen D, Xia X, Wang H, Luo Y, Barceló D (2020) Response of soil enzyme activities and bacterial communities to the accumulation of microplastics in an acid cropped soil. Sci Total Environ 707:135634. 10.1016/j.scitotenv.2019.135634 31761364 10.1016/j.scitotenv.2019.135634

[CR17] Fu L, Li J, Wang G, Luan Y, Dai W (2021) Adsorption behavior of organic pollutants on microplastics. Ecotoxicol Environ Saf 217:112207. 10.1016/j.ecoenv.2021.112207 33866287 10.1016/j.ecoenv.2021.112207

[CR18] Gatidou G, Arvaniti OS, Stasinakis AS (2019) Review on the occurrence and fate of microplastics in sewage treatment plants. J Hazard Mater 367:504–512. 10.1016/j.jhazmat.2018.12.081 30620926 10.1016/j.jhazmat.2018.12.081

[CR19] Gies EA, Lenoble JL, Noël M, Etemadifar A, Bishay F, Hall ER, Ross PS (2018) Retention of microplastics in a major secondary wastewater treatment plant in Vancouver, Canada. Mar Pollut Bull 133:553–561. 10.1016/j.marpolbul.2018.06.006 30041349 10.1016/j.marpolbul.2018.06.006

[CR20] Guo J, Huang XP, Xiang L, Wang YZ, Li YW, Li H, Cai QY, Mo CH, Wong MH (2020) Source, migration and toxicology of microplastics in soil. Environ Int 137:105263. 10.1016/j.envint.2019.105263 32087481 10.1016/j.envint.2019.105263

[CR21] Hakanson L (1980) An ecological risk index for aquatic pollution control. A sedimentological approach. Water Res 14(8):975–1001. 10.1016/0043-1354(80)90143-8

[CR22] Hattab S, Boughattas I, Alaya C, Gaaied S, Romdhani I, El Gaied F, Abouda S, Mokni M, Banni M (2024) Assessing the presence of microplastic in agriculture soils irrigated with treated waste waters using Lumbricus sp.: Ecotoxicological effects. Sci Total Environ 950:175096. 10.1016/j.scitotenv.2024.175096 39079648 10.1016/j.scitotenv.2024.175096

[CR23] He D, Luo Y, Lu S, Liu M, Song Y, Lei L (2018) Microplastics in soils: Analytical methods, pollution characteristics and ecological risks. TrAC. Trends Anal Chem 109:163–172. 10.1016/j.trac.2018.10.006

[CR24] Hossain A, Adham MI, Hasan M, Ali MM, Siddique MAB, Senapathi V, Islam ARMT (2024) Analysis and risk evaluation of soil microplastics in the Rohingya refugee camp area, Bangladesh: A comprehensive study. Reg Stud Mar Sci 76:103578. 10.1016/j.rsma.2024.103578

[CR25] Huang Y, Liu Q, Jia W, Yan C, Wang J (2020) Agricultural plastic mulching as a source of microplastics in the terrestrial environment. Environ Pollut 260:114096. 10.1016/j.envpol.2020.114096 32041035 10.1016/j.envpol.2020.114096

[CR26] Islam T, Cheng H (2024) Existence and fate of microplastics in terrestrial environment: A global fretfulness and abatement strategies. Sci Total Environ 953:176163. 10.1016/j.scitotenv.2024.176163 39260485 10.1016/j.scitotenv.2024.176163

[CR27] ISO 11464 (2006) Soil quality - Pretreatment of samples for physico-chemical analysis. International Organization for Standardization, Geneva

[CR28] Jackson ML (1962) Soil Chemical Analysis. Prentice-Hall, Inc., Englewood Cliffs., NJ

[CR29] Jia Z, Wei W, Wang Y, Chang Y, Lei R, Che Y (2024) Occurrence characteristics and risk assessment of microplastics in agricultural soils in the loess hilly gully area of Yan’ an. China Sci Total Environ 912:169627. 10.1016/j.scitotenv.2023.169627 38157894 10.1016/j.scitotenv.2023.169627

[CR30] Jiang XJ, Liu W, Wang E, Zhou T, Xin P (2017) Residual plastic mulch fragments effects on soil physical properties and water flow behavior in the Minqin Oasis, northwestern China. Soil Tillage Res 166:100–107. 10.1016/j.still.2016.10.011

[CR31] Jiang X, Chen H, Liao Y, Ye Z, Li M, Klobucar G (2019) Ecotoxicity and genotoxicity of polystyrene microplastics on higher plant Vicia faba. Environ Pollut 250:831–838. 10.1016/j.envpol.2019.04.055 31051394 10.1016/j.envpol.2019.04.055

[CR32] Kang H, Yu W, Dutta S, Gao H (2021) Soil microbial community composition and function are closely associated with soil organic matter chemistry along a latitudinal gradient. Geoderma 383:114744. 10.1016/j.geoderma.2020.114744

[CR33] Karthick V, Siddhuraju P (2024) Identification and characterization of microplastics contamination in domestic sewage wastewater irrigated of Napier grass (Pennisetum purpureum) agricultural field. Proc Indian Natl Sci Acad 90:1074–1083. 10.1007/s43538-024-00298-7

[CR34] Khalid N, Aqeel M, Noman A, Khan SM, Akhter N (2021) Interactions and effects of microplastics with heavy metals in aquatic and terrestrial environments. Environ Pollut 290:118104. 10.1016/j.envpol.2021.118104 34500399 10.1016/j.envpol.2021.118104

[CR35] Khan AR, Ulhassan Z, Li G, Lou J, Iqbal B, Salam A, Azhar W, Batool S, Zhao T, Li K, Zhang Q, Zhao X, Du D (2024) Micro/nanoplastics: critical review of their impacts on plants, interactions with other contaminants (antibiotics, heavy metals, and polycyclic aromatic hydrocarbons), and management strategies. Sci Total Environ 912:169420. 10.1016/j.scitotenv.2023.169420 38128670 10.1016/j.scitotenv.2023.169420

[CR36] Lan ZL, Zhang SL, Sial TA, Wu L, Chang W, Li X, Zhang J, Fan J (2022) Fine-scale spatial distribution of soil organic carbon and its fractions after afforestation withPinus sylvestrisandSalix psammophilain a semiarid desert of China. J Plant Ecol 15:141–154. 10.1093/jpe/rtab078

[CR37] Lehmann A, Zheng W, Rillig MC (2017) Soil biota contributions to soil aggregation. Nat Ecol Evol 1(12):1828–1835. 10.1038/s41559-017-0344-y 29038473 10.1038/s41559-017-0344-y PMC5701735

[CR38] Lei L, Liu M, Song Y, Lu S, Hu J, Cao C, Xie B, Shi H, He D (2018) Polystyrene (nano) microplastics cause size-dependent neurotoxicity, oxidative damage and other adverse effects in Caenorhabditis elegans. Environ Sci: Nano 5:2009–2020. 10.1039/c8en00412a

[CR39] Li X, Chen L, Mei Q, Dong B, Dai X, Ding G, Zeng EY (2018) Microplastics in sewage sludge from the wastewater treatment plants in China. Water Res 142:75–85. 10.1016/j.watres.2018.05.034 29859394 10.1016/j.watres.2018.05.034

[CR40] Li L, Luo Y, Li R, Zhou Q, Peijnenburg WJGM, Yin N, Yang J, Tu C, Zhang Y (2020) Effective uptake of submicrometre plastics by crop plants via a crack-entry mode. Nat Sustainability 3:929–937. 10.1038/s41893-020-0567-9

[CR41] Li J, Guo K, Cao Y, Wang S, Song Y, Zhang H (2021) Enhance in mobility of oxytetracycline in a sandy loamy soil caused by the presence of microplastics. Environ Pollut 269:116151. 10.1016/j.envpol.2020.116151 33280909 10.1016/j.envpol.2020.116151

[CR42] Liang Y, Lehmann A, Yang G, Leifheit EF, Rillig MC (2021) Effects of microplastic fibers on soil aggregation and enzyme activities are organic matter dependent. Front Environ Sci 9:650155. 10.3389/fenvs.2021.650155

[CR43] Lithner D, Larsson Å, Dave G (2011) Environmental and health hazard ranking and assessment of plastic polymers based on chemical composition. Sci Total Environ 409:3309–3324. 10.1016/j.scitotenv.2011.04.038 21663944 10.1016/j.scitotenv.2011.04.038

[CR44] Liu H, Yang X, Liu G, Liang C, Xue S, Chen H, Ritsema CJ, Geissen V (2017) Response of soil dissolved organic matter to microplastic addition in Chinese loess soil. Chemosphere 185:907–917. 10.1016/j.chemosphere.2017.07.064 28747000 10.1016/j.chemosphere.2017.07.064

[CR45] Luo Y, Zhang Y, Xu Y, Guo X, Zhu L (2020) Distribution characteristics and mechanism of microplastics mediated by soil physicochemical properties. Sci Total Environ 726:138389. 10.1016/j.scitotenv.2020.138389 32305754 10.1016/j.scitotenv.2020.138389

[CR46] Majewsky M, Bitter H, Eiche E, Horn H (2016) Determination of microplastic polyethylene (PE) and polypropylene (PP) in environmental samples using thermal analysis (TGA-DSC). Sci Total Environ 568:507–511. 10.1016/j.scitotenv.2016.06.017 27333470 10.1016/j.scitotenv.2016.06.017

[CR47] Mason SA, Garneau D, Sutton R, Chu Y, Ehmann K, Barnes J, Fink P, Papazissimos D, Rogers DL (2016) Microplastic pollution is widely detected in US municipal wastewater treatment plant effluent. Environ Pollut 218:1045–1054. 10.1016/j.envpol.2016.08.056 27574803 10.1016/j.envpol.2016.08.056

[CR48] Masura J, Baker J, Foster G, Arthur C, Herring C, Editor T (2015) Laboratory methods for the analysis of microplastics in the marine environment: recommendations for quantifying synthetic particles in waters and sediments. NOAA Technical Memorandum NOS-OR&R-48. 10.25607/OBP-604

[CR49] Mbachu O, Jenkins G, Kaparaju P, Pratt CJ (2021) The rise of artificial soil carbon inputs: reviewing microplastic pollution effects in the soil environment. Sci Total Environ 780:146569. 10.1016/j.scitotenv.2021.146569 33770603 10.1016/j.scitotenv.2021.146569

[CR50] Nannipieri P, Eldor P (2009) The chemical and functional characterization of soil N and its biotic components. Soil Biol Biochem 41:2357–2369. 10.1016/j.soilbio.2009.07.013

[CR51] Nelson DW, Sommers LE (1982) Total carbon, organic carbon, and organic matter. In: Page AL, Miller RH, Kenney DR (eds) Methods of Soil Analysis, Part 2, Am Soc Agron Madison, pp 539–579. 10.2134/agronmonogr9.2.2ed.c29

[CR52] Nizzetto L, Langaas S, Futter M (2016) Pollution: do microplastics spill on to farm soils? Nature 537:488. 10.1038/537488b. (**ISSN 1476-4687**) 10.1038/537488b 27652556

[CR53] Olsen SR, Cole CV, Watanabe FS (1954) Estimation of Available Phosphorus in Soils by Extraction with Sodium Bicarbonate. USDA Circular No. 939, US Government Printing Office, Washington DC

[CR54] Palansooriya KN, Shi L, Sarkar B, Parikh SJ, Sang MK, Lee SR, Ok YS (2022) Effect of LDPE microplastics on chemical properties and microbial communities in soil. Soil Use Management 38(3):1481–1492. 10.1111/sum.12808

[CR55] Qi Y, Yang X, Pelaez AM, Huerta Lwanga E, Beriot N, Gertsen H, Garbeva P, Geissen V (2018) Macro- and micro- plastics in soil-plant system: effects of plastic mulch film residues on wheat (Triticum aestivum) growth. Sci Total Environ 645:1048–1056. 10.1016/j.scitotenv.2018.07.229 30248830 10.1016/j.scitotenv.2018.07.229

[CR56] Qi Y, Ossowicki A, Yang X, Lwanga EH, Dini-Andreote F, Geissen V, Garbeva P (2020) Effects of plastic mulch film residues on wheat rhizosphere and soil properties. J Hazard Mater 387:121711. 10.1016/j.jhazmat.2019.121711 31806445 10.1016/j.jhazmat.2019.121711

[CR57] Raju S, Carbery M, Kuttykattil A, Senathirajah K, Subashchandrabose SR, Evans G, Thavamani P (2018) Transport and fate of microplastics in wastewater treatment plants: implications to environmental health. Rev Environ Sci Bio/technol 17:637–653. 10.1007/s11157-018-9480-3

[CR58] Ranjani M, Veerasingam S, Venkatachalapathy R, Mugilarasan M, Bagaev A, Mukhanov V, Vethamony PJMPB (2021) Assessment of potential ecological risk of microplastics in the coastal sediments of India: a meta-analysis. Mar Pollut Bull 163:111969. 10.1016/j.marpolbul.2021.111969 33515857 10.1016/j.marpolbul.2021.111969

[CR59] Richard LA (1954) Diagnosis and Improvement of Saline and Alkali Soils. US Department of Agriculture. Agricultural Handbook No. 60, Washington DC, pp 7–53. 10.1097/00010694-195408000-00012.

[CR60] Rillig MC, Lehmann A (2020) Microplastic in terrestrial ecosystems. Science 368(6498):1430–1431. 10.1126/science.abb5979 32587009 10.1126/science.abb5979 PMC7115994

[CR61] Rillig MC, Ingraffia R, de Souza Machado AA (2017) Microplastic incorporation into soil in agroecosystems. Front Plant Sci 8:1805. 10.3389/fpls.2017.01805 29093730 10.3389/fpls.2017.01805 PMC5651362

[CR62] Rilling MC (2012) Microplastic in Terrestrial Ecosystems and the Soil? Environ Sci Technol 46(12):6453–6454. 10.1021/es302011r 22676039 10.1021/es302011r

[CR63] Rodrigues MO, Gonçalves AMM, Gonçalves FJM, Nogueira H, Marques JC, Abrantes N (2018) Efectiveness of a methodology of microplastics isolation for environmental monitoring in freshwater systems. Ecol Indic 89:488–495. 10.1016/j.ecolind.2018.02.038

[CR64] Rong L, Zhao L, Zhao L, Cheng Z, Yao Y, Yuan C, Wang L, Sun H (2021) LDPE microplastics affect soil microbial communities and nitrogen cycling. Sci Total Environ 773:145640. 10.1016/j.scitotenv.2021.145640 33582358 10.1016/j.scitotenv.2021.145640

[CR65] Salehi Z, Hashemi SH, Flury M (2023) Micro- and Mesoplastics in Farmlands with Different Irrigation Water Sources. Water Air Soil Pollut 234(4). 10.1007/s11270-023-06289-6

[CR66] Scheurer M, Bigalke M (2018) Microplastics in Swiss floodplain soils. Environ Sci Technol 52:3591–3598. 10.1021/acs.est.7b06003 29446629 10.1021/acs.est.7b06003

[CR67] Shen M, Song B, Zhou C, Almatrafi E, Hu T, Zeng G, Zhang Y (2022) Recent advances in impacts of microplastics on nitrogen cycling in the environment: a review. Sci Total Environ 815:152740. 10.1016/j.scitotenv.2021.152740 34974017 10.1016/j.scitotenv.2021.152740

[CR68] Siddiqui MN, Gondal MA, Redhwi HH (2008) Identification of different type of polymers in plastics waste. J Environ Sci Health Part A Toxic/hazard Subst Environ Eng 43(11):1303–1310. 10.1080/10934520802177946 10.1080/10934520802177946 18642154

[CR69] Spohn M (2020) Phosphorus and carbon in soil particle size fractions: a synthesis. Biogeochemistry 147:225–242. 10.1007/s10533-019-00633-x

[CR70] Wang L, Peng Y, Xu Y, Zhang J, Liu C, Tang X, Lu Y, Sun H (2022) Earthworms’ degradable bioplastic diet of polylactic acid: easy to break down and slow to excrete. Environ Sci Technol 56:5020–5028. 10.1021/acs.est.1c08066 35383459 10.1021/acs.est.1c08066

[CR71] Yan Y, Chen Z, Zhu F, Zhu C, Wang C, Gu C (2020) Effect of polyvinyl chloride microplastics on bacterial community and nutrie status in two agricultural soils. B Environ Contam Tox 106:236–244. 10.1007/s00128-020-02963-1 10.1007/s00128-020-02963-1 32813033

[CR72] Yang L, Zhang Y, Kang S, Wang Z, Wu C (2021) Microplastics in soil: A review on methods, occurrence, sources, and potential risk. Sci Total Environ 780:146546. 10.1016/j.scitotenv.2021.146546 33770602 10.1016/j.scitotenv.2021.146546

[CR73] Yang X, Wan Z, Xiao J, Li F, Zhang F, Zhang Z (2024) Evaluation of niche, diversity, and risks of microplastics in farmland soils of different rocky desertification areas. J Hazard Mater 466:133603. 10.1016/j.jhazmat.2024.133603 38280320 10.1016/j.jhazmat.2024.133603

[CR74] Yi M, Zhou S, Zhang L, Ding S (2021) The effects of three different microplastics on enzyme activities and microbial communities in soil.Water Environ. Res 93:24–32. 10.1002/wer.1327 10.1002/wer.1327 32187766

[CR75] Yin L, Wen X, Huang D, Du C, Deng R, Zhou Z, Tao J, Li R, Zhou W, Wang Z, Chen H (2021) Interactions between microplastics/nanoplastics and vascular plants. Environ Pollut 290:117999. 10.1016/j.envpol.2021.117999 34500397 10.1016/j.envpol.2021.117999

[CR76] Zhang GS, Liu YF (2018) The distribution of microplastics in soil aggregate fractions in southwestern China. Sci Total Environ 642:12–20. 10.1016/j.scitotenv.2018.06.004 29894871 10.1016/j.scitotenv.2018.06.004

[CR77] Zhang GS, Zhang FX, Li XT (2019) Effects of polyester microfibers on soil physical properties: perception from a field and a pot experiment. Sci Total Environ 670:1–7. 10.1016/j.scitotenv.2019.03.149 30893616 10.1016/j.scitotenv.2019.03.149

[CR78] Zhang P, Peng H, Hongwen S, Beixing JM, L, (2020) The structure of agricultural microplastics (PT, PU and UF) and their sorption capacities for PAHs and PHE derivates under various salinity and oxidation treatments. Environ Pollut 257:113525. 10.1016/j.envpol.2019.113525 31761592 10.1016/j.envpol.2019.113525

[CR79] Zhang Y, Wang K, Chen W, Ba Y, Khan K, Chen W, Tu C, Chen C, Xu L (2022) Effects of land use and landscape on the occurrence and distribution of microplastics in soil, China. Sci Total Environ 847:157598. 10.1016/j.scitotenv.2022.157598 35878858 10.1016/j.scitotenv.2022.157598

[CR80] Zhang F, Yang X, Zhang Z (2024) Effects of soil properties and land use patterns on the distribution of microplastics: A case study in southwest China. J Environ Manage 356:120598. 10.1016/j.jenvman.2024.120598 38490007 10.1016/j.jenvman.2024.120598

[CR81] Zhou B, Wang J, Zhang H, Shi H, Fei Y, Huang S, Tong Y, Wen D, Luo Y, Barceló D (2020) Microplastics in agricultural soils on the coastal plain of Hangzhou Bay, east China: Multiple sources other than plastic mulching film. J Hazard Mater 388:121814. 10.1016/j.jhazmat.2019.121814 31843412 10.1016/j.jhazmat.2019.121814

[CR82] Zhou J, Wen Y, Marshall MR, Zhao J, Gui H, Yang Y, Zeng Z, Jones DL, Zang H (2021) Microplastics as an emerging threat to plant and soil health in agroecosystems. Sci Total Environ 787:147444. 10.1016/j.scitotenv.2021.147444

[CR83] Zhu D, Bi Q, Xiang Q, Chen Q, Christie P, Ke X, Wu L, Zhu Y (2018) Trophic predator-prey relationships promote transport of microplastics compared with the single Hypoaspis aculeifer and Folsomia candida. Environ Pollut 235:150–154. 10.1016/j.envpol.2017.12.058 29284144 10.1016/j.envpol.2017.12.058

[CR84] Zhu F, Yan Y, Doyle E, Zhu C, Jin X, Chen Z, Wang C, He H, Zhou D, Gu C (2022) Microplastics altered soil microbiome and nitrogen cycling: the role of phthalate plasticizer. J Hazard Mater 427:127944. 10.1016/j.jhazmat.2021.127944 34865900 10.1016/j.jhazmat.2021.127944

[CR85] Ziajahromi S, Neale PA, Rintoul L, Leusch FDL (2017) Wastewater treatment plants as a pathway for microplastics: development of a new approach to sample wastewater-based microplastics. Water Res 112:93. 10.1016/j.watres.2017.01.042 28160700 10.1016/j.watres.2017.01.042

